# Supplementary material for: Prevalence and causes of ocular disorders and visual impairment among preterm children in Ethiopia
Source: BMJ Paediatr Open. 2024 Feb 7;8(1):e002317. doi: 10.1136/bmjpo-2023-002317 (PMC10860044; doi:10.1136/bmjpo-2023-002317)
Supplement: Author's manuscript [file bmjpo-2023-002317.draft_revisions.pdf]

BMJ Paediatrics Open

BMJ Paediatrics Open is committed to open peer review. As part of this commitment we make the peer review history of every article we publish publicly available.

When an article is published we post the peer reviewers' comments and the authors' responses online. We also post the versions of the paper that were used during peer review. These are the versions that the peer review comments apply to.

The versions of the paper that follow are the versions that were submitted during the peer review process. They are not the versions of record or the final published versions. They should not be cited or distributed as the published version of this manuscript.

BMJ Paediatrics Open is an open access journal and the full, final, typeset and author-corrected version of record of the manuscript is available on our site with no access controls, subscription charges or pay-per-view fees (http://bmjpaedsopen.bmj.com).

If you have any questions on BMJ Paediatrics Open's open peer review process please email info.bmjpo@bmj.com

BMJ Paediatrics Open

Prevalence and causes of ocular disorders and visual impairment among preterm children in Ethiopia

Journal:	*BMJ Paediatrics Open*
Manuscript ID	bmjpo-2023-002317
Article Type:	Original research
Date Submitted by the Author:	05-Oct-2023
Complete List of Authors:	SHERIEF, SADIK; Addis Ababa University, Department of Ophthalmology; SickKids Research Institute<br>Muhe, Lulu M; Addis Ababa University College of Health Sciences<br>Mekasha, Amha; Addis Ababa University, Department of Pediatrics and Child Health<br>Demtse, Asrat; Addis Ababa University College of Health Sciences, Paediatrics and Child Health<br>Ali, Asim; The Hospital for Sick Children, Ophthalmology and Vision Sciences
Keywords:	Infant, Neonatology, Ophthalmology

SCHOLARONE™
Manuscripts

I, the Submitting Author has the right to grant and does grant on behalf of all authors of the Work (as defined in the below author licence), an exclusive licence and/or a non-exclusive licence for contributions from authors who are: i) UK Crown employees; ii) where BMJ has agreed a CC-BY licence shall apply, and/or iii) in accordance with the terms applicable for US Federal Government officers or employees acting as part of their official duties; on a worldwide, perpetual, irrevocable, royalty-free basis to BMJ Publishing Group Ltd ("BMJ") its licensees and where the relevant Journal is co-owned by BMJ to the co-owners of the Journal, to publish the Work in this journal and any other BMJ products and to exploit all rights, as set out in our licence.

The Submitting Author accepts and understands that any supply made under these terms is made by BMJ to the Submitting Author unless you are acting as an employee on behalf of your employer or a postgraduate student of an affiliated institution which is paying any applicable article publishing charge ("APC") for Open Access articles. Where the Submitting Author wishes to make the Work available on an Open Access basis (and intends to pay the relevant APC), the terms of reuse of such Open Access shall be governed by a Creative Commons licence – details of these licences and which Creative Commons licence will apply to this Work are set out in our licence referred to above.

Other than as permitted in any relevant BMJ Author's Self Archiving Policies, I confirm this Work has not been accepted for publication elsewhere, is not being considered for publication elsewhere and does not duplicate material already published. I confirm all authors consent to publication of this Work and authorise the granting of this licence.

Confidential: For Review Only

Prevalence and causes of ocular disorders and visual impairment among preterm children in Ethiopia

Sadik Taju Sherief [1,2], Lulu Muhe [3] Amha Mekasha [3], Asrat Demtse [3], and Asim Ali[4]

[1] Department of Ophthalmology, Addis Ababa University, Addis Ababa, Ethiopia;

[2] Child Health Evaluative Sciences Program and Centre for Global Child Health, Sickkids Research Institute, Toronto, Canada;

[3] Department of Pediatrics and Child Health, Addis Ababa University, Addis Ababa, Ethiopia;
[4] Department of Ophthalmology and Vision Sciences, The Hospital for Sick Children and University of Toronto, Toronto, Canada.

Short Title: Ocular disorders in Preterms

Corresponding Author:
Sadik T. Sherief, MD
Department of Ophthalmology
School of Medicine, College of Health Sciences
Addis Ababa University
P.O. Box 9086, Addis Ababa, Ethiopia
Tel: +251917801719
Email: goge4000@yahoo.com

Submitted as an Original Research article to: BMJ Paediatrics Open

Abstract word count (300 maximum): 235 words

Manuscript word count (4000 maximum): 3,433 words

Tables and Figures (maximum 5): 4 Tables

Reference Count: 54

Submitted to: *BMJ Paediatrics Open*

Key words: Preterm children; Low birth weight; Visual Impairment; Refractive error; Strabismus; Retinopathy of prematurity; Risk factors of visual impairment; Sub-Saharan Africa; Ethiopia.

Confidential: For Review Only

ABSTRACT

Objective

The aim of this study was to determine the prevalence, causes of ocular disorders and visual impairment among preterm children previously admitted to neonatal intensive care units in Addis Ababa, Ethiopia.

Methods and Analysis

A prospective screening survey was conducted from Feb. to June 2019 at the pediatric eye clinic of Menelik II Hospital. Children who were preterm at birth and who attended the eye clinic were included in the study. Data on demographic and neonatal characteristics, neonatal and maternal co-morbidities, and ocular disorders were collected. Odds ratio and univariate analysis were used to identify predictors of ocular diseases and visual impairment.

Results

There were 222 children included in the study with a mean age at presentation of 2.62 years (range 2.08- 6.38 years), mean GA 34.11 weeks (range 30-36) weeks, and mean birthweight 1941.72g (range 953-3500g). Nearly 2/3 had ocular disorders with refractive error (51.8%), strabismus (11.3%), and history of ROP (7.2%) being more common. One-fourth of the children had visual impairment, and the prevalence of amblyopia was 40.1%. Uncorrected refractive errors, strabismus, and ROP were causes for visual impairment.

Conclusion

Visual impairment and amblyopia are common in Ethiopia. There is a need to develop a screening protocol for ocular disorders for preterm children to enhance early detection and prevention of childhood visual impairment.

Keywords: Preterm children; Low birth weight; Visual Impairment; Refractive error; Strabismus; Retinopathy of prematurity; Risk factors of visual impairment; Sub-Saharan Africa; Ethiopia.

Confidential: For Review Only

Key Messages

What is already known about this subject?

- In many low- and middle-income countries, the survival of preterm infants has improved as neonatal systems have improved.
- Preterm children are at a higher risk of developing ocular disorders, visual impairment, and amblyopia than term children.

What this study adds

- The magnitude and causes of ocular morbidity among preterm children are not well studied in sub-Saharan African countries. This study, conducted among preterm children admitted to two NICUs in a sub-Saharan country, shows that preterm infants develop a higher rate of visual impairment and amblyopia.

How this study might affect research, practice or policy

- The findings of this study provide some evidence for screening for ocular diseases in preterm children, but further studies are needed.

Confidential: For Review Only

INTRODUCTION

Global, regional, and national estimates of preterm birth (defined as childbirth at less than 37 completed weeks) using the 2019 Global Burden of Disease study showed 15.22 million preterm births (1). In the Global Burden of Disease Study, 3.1% of all disability-adjusted life-years were attributed to preterm birth, similar to the burden of HIV or malaria (2). More than 95% of preterm births are occurring in developing countries. Globally the estimated preterm birth rate is 11.1%. Over 60% of preterm births occur in Sub-Saharan Africa and South Asia (1). Ethiopia belongs to the top 15 countries that contribute to two-thirds of the world's preterm babies with a preterm rate of 14.1% out of 481 deliveries (3).

From six months of pregnancy to term is considered the most active period for ocular development (4). Improved neonatal care has increased the survival rates of extremely preterm infants with birth weights (BW) of 1,000 g or gestational age (GA) of 28 weeks; at the same time, retinopathy of prematurity (ROP) has become a significant threat to visual function (5-7). Preterm children are reported to have an increased incidence of visual impairment because of perinatal lesions in the brain (8-10).

It has been noted that both preterm birth and retinopathy of prematurity (ROP) have an effect on the developing visual system, leading to decreased visual acuity, decreased contrast sensitivity, and an increase in color vision deficiencies (11-16). Population-based studies suggest that ophthalmic impairments remain common in very low birth weight infants (11,16,17). Effects of prematurity on ocular and neurological development include retinopathy of prematurity (ROP), refractive error, strabismus, cerebral visual impairment, color vision deficits, reduced contrast sensitivity (CS), visual field defects, and decreased visual acuity (16). According to population studies, the incidence of ROP, whether moderate or severe, for infants born at less than 1500–1700 g ranges from 22–49% (17-19).

In a cohort study, children with lower birth weights had significantly worse near and distance visual acuity at ages 10 to 12 years compared to full-term infants (10). Additionally, infants born prematurely without ROP are more likely to have myopia and anisometropia than infants born at term because preterm babies are more likely to experience refractive errors (20). An increased incidence of strabismus has also been reported in children born prematurely,

Confidential: For Review Only

regardless of the presence of ROP (21-24).

Research on ocular morbidities among preterm infants in sub-Saharan African nations is limited. Before 2020, blindness from ROP was not reported in Ethiopia, including studies in schools for the blind (25, 26).

To determine the top causes of illness and mortality in preterm infants admitted to neonatal intensive units (NICUs) in Ethiopia, an Ethiopian Study of Illness in Preterms (SIP) study was conducted based on standardized diagnostic protocols. This study is part of the SIP study focusing on ocular morbidities among preterms. The present study aimed to identify ocular disorders in a population of preterm children with and without ROP.

METHODS

Study design and subjects

The SIP Study is a prospective study conducted to determine the top causes of illness and mortality in preterm infants admitted to hospitals in Ethiopia based on standardized diagnostic protocols (27). The study participants of this current study are from the SIP study from Feb – June, 2019. The research was performed in accordance with the Declaration of Helsinki and was approved by the Institute Ethics Committee of Addis Ababa University ((Ref No. 003/2016). All parents or legal guardians provided informed consent before the examination. Patients or the public weren't involved in our research's design, conduct, reporting, or dissemination plans.

Study setting

For the SIP study, standard protocols were developed to undertake a physical examination and laboratory investigation, in particular microbiology, radiologic, and ultrasound examinations. There were initial and follow-up examinations to detect the progress of the preterm infant. Addis Ababa University, Gondar University, Jimma University, and St. Paul Millennium Medical College were included in the SIP study. However, for this ocular morbidity aspect of the SIP study, preterms from Addis Ababa, Tikur Anbessa Hospital, Gandhi Hospital, and St. Paul Millennium Medical College were included in the research.

Recruitment methods

Inclusion criteria were (1) GA < 37 weeks and (2) participation in the SIP study. The preterm children were identified from the SIP database. Parents of all preterm infants received a phone call invitation to participate in our investigation.

Confidential: For Review Only

Confidential: For Review Only

Assessment of prenatal and postnatal history

History data were assessed from each child's recorded file for the enrolled children. The following data were extracted: antenatal risk factors: maternal age, in vitro fertilization, antenatal corticosteroids, preeclampsia/eclampsia, diabetes, HIV/AIDS, chorioamnionitis, mode of delivery, and multiple births. Neonatal factors included sex, GA, BW, resuscitation in the delivery room, respiratory distress syndrome (RDS), duration of invasive/noninvasive mechanical ventilation and oxygen therapy, intracranial hemorrhage, patent ductus arteriosus (PDA), neonatal sepsis, necrotizing enterocolitis (NEC), number of blood transfusions, and bronchopulmonary dysplasia (BPD). There were no regular ROP screening programmes within the NICUs of the hospitals where the patients were admitted. There was no referral system from the NICUs to Ophthalmology clinic, except if the parents noted a concern. In addition, all parents were interviewed using a standardized protocol to request information concerning medical history of the child and parents, including ocular and general morbidities.

Definitions

Gestational age was determined using last menstrual period [LPM], Ballard and Dubowitz scores and ultrasound assessment. Studies in Papua New Guinea have shown good concordance (0.878, 0.914, and 0.886, respectively) compared to antenatal ultrasound as the gold standard (28). LMP in a low-resource setting such as Bangladesh was found to be a more reliable measure of gestational age than previously thought for the estimation of postnatal gestational age of preterm infants (29).

Preterm infants were further classified as late and moderate preterm (32 to < 37 weeks), very preterm (28 to <32 weeks), and extremely preterm (less than 28 weeks). Glasses were prescribed if there was myopia >1.0D, astigmatism >1.0D, or hypermetropia >+2.0D.

Eye examination

All examinations were performed by the PI and lead author (STS), a pediatric ophthalmologist. Testing of best-corrected visual acuity was performed with Lea symbols until school enrolment, and after that, ETDRS was used in all subjects. In cases of visual acuity below 6/60, depending on the children's age, Lea symbols or Landolt rings were used at a distance of 1 m. Values were converted for analysis into the logarithm of the minimum angle of resolution (logMAR) (30).

Cyclopentolate (0.5%) eye drops were administered three times at 10-min intervals, after which cycloplegic refraction and keratometry were analyzed with an autorefractor (Nidek ARK-1s keratometer, Japan). The spherical equivalent (refractive error) was calculated by adding the spherical value and half of the cylindrical value. Anisometropia was defined as a difference between the patients' eyes of ≥ 1.5 diopters of spherical equivalent. Orthoptic examination for strabismus included the cover-uncover test and alternate cover test, the Hirschberg Test and examination of fixation behavior, as well as the presence or absence of nystagmus after having corrected refractive errors. If a child presented with heterotropia, an alternating prism cover test was added to measure the angle of deviation in prism diopters.

Strabismus was defined as constant or intermittent heterotropia of any dimension at a distance and/or near fixation after correcting refractive error. Classification of strabismus was categorized depending on deviation from the primary position (esotropia or exotropia). An anterior segment examination was done using slit lamp biomicroscopy. A dilated posterior segment examination was conducted using indirect ophthalmoscopy with a 28-diopter lens. Retinopathy of prematurity was diagnosed retrospectively from the patients' chart.

Statistical analyses

Statistical analysis was undertaken using IBM SPSS 21.0 (SPSS Inc., Chicago, USA). Continuous variables were expressed as the mean ± standard deviation (SD) or as the median when appropriate. Categorical variables were expressed as proportions. The chi-square test was used to analyze the association between categorical variables. Associations between ocular morbidities and continuous and categorical variables were computed using Fisher's exact test and Pearson chi-square (χ^2) test, respectively. Continuous variables were compared using ANOVA. Values of $p < 0.05$ were considered statistically significant.

Two statistical models were used for risk factor analysis. First, separate univariate logistic regression analysis was performed with the presence of ocular morbidities as a dependent variable and documented potential risk factors for ocular morbidities as independent variables. Second, variables that were significant at the 0.25 level in univariable analysis were used in the multivariable mode. The goodness of fit of the final model was assessed using the Hosmer and Lemeshow test (31). Adjusted odds ratios are reported with 95% confidence-intervals; a p-value of <0.05 was considered statistically significant.

For peer review only

RESULTS

During the study period 222 infants (146 from Saint Paul Hospital and 76 from TASH) were included in this study.

Characteristics of the study population

Slightly more females than males were screened (52.7% and 47.3%, respectively). The majority of the study participants (n=156, 70.3%) were less than 3 years of age and the mean age at presentation was 2.62 ± 0.49 years (range 2.08- 6.38). One hundred and twenty-three of the 222 children (55.4%) had a GA ≤34 weeks and 43 (19.4%) had a BW ≤1500g. Birthweight ranged from 953-3500g with a mean of 1941.72g (SD 445.49); GA ranged from 30-36 weeks, with a mean of 34.11 weeks (SD 1.47). One hundred and twenty-three children (55.4%) were delivered vaginally, and 80 (36.1%) had multiple gestations. Forty-eight children (21.7%) were born to mothers with pregnancy-induced hypertension, and eight (3.7%) mothers tested positive for HIV (Table 1).

The mean BWs of children from SPH and TASH NICUs were 1888.5 ± 403.6 (953-3000) g and 2043.94 ± 503.74 (1125-3500) g, respectively; mean GAs were 34.14 ± 1.49 (30-36) weeks and 34.08 ± 1.44 (30-36) weeks, respectively. Differences in these parameters were not statistically significant (Table 2).

Confidential: For Review Only

Table 1 Characteristics of premature children and mothers screened for ocular disorders

Variable		Total	Male		Female	
		N	N	%	N	%
**Birthweight**	≤1500 gm	43	20	46.5%	23	53.5%
	>1500gm	179	85	47.5%	94	52.5%
**Gestational age**	≤34 weeks	122	60	49.2%	62	50.8%
	> 34 weeks	100	45	45%	55	55%
**Multiple gestation**	Yes	80	42	52.5%	38	47.5%
	No	142	63	44.4%	79	55.6%
**Oxygen supplementation**	Yes	97	47	48.5%	50	51.5%
	No	125	58	46.4%	67	53.6%
**Infantile morbidity**	Sepsis	6	2	33.3%	4	66.7%
	IVH	2	0	0%	2	100%
	BPD and Sepsis	1	1	100%	0	0%
	None	213	102	47.9%	111	52.1%
**Mode of delivery**	Vaginal delivery	123	53	43.1%	70	56.9%
	Cesarean section	99	52	52.5%	47	47.5%
**Multiparity**	Yes	47	18	38.3%	29	61.7%
	No	175	63	34.3	59	65.7%
**Maternal morbidity**	PIH	44	22	50%	22	50%
	HIV	5	2	40%	3	60%
	HIV & PIH	3	3	100%	0	0%
	DM	2	0	0%	2	100%
	DM & PIH	1	0	0%	1	100%
	TORCH	1	1	100%	0	0%
	None	166	77	46.4%	89	53.6%
**NICU location**	SPH	146	65	44.5%	81	55.5%
	TASH	76	40	52,6%	36	47.4%

Legend: BPD- Bronchopulmonary Dysplasia; DM- Diabetes Mellitus; HIV-IVH-Intraventricular Hemorrhage; PIH- Pregnancy-Induced Hypertension; SPH- Saint Paul Hospital; TASH-Tikur Anbessa Specialized Hospital; TORCH- Toxoplasmosis, Rubella, Cytomegalovirus, Herpes simplex, and HIV

Ocular morbidities and risk factors

Overall, 145 (65.3%) of the children had ocular disorders at the presentation, of which 92 (63.4%) had isolated ocular diseases (69 refractive error, 13 nasolacrimal duct obstruction, five strabismus, and five ROP). The mean age at presentation of children with ocular disorders was 2.7±0.5 (2.1- 6.4) years, and there were more females with a male to female ratio of 1:1.27. None of the eyes examined had anomalies of the anterior segment or lens.

The mean GA was 34.14 ± 1.49 (30-36) weeks, and BW was 1927.27 ± 429.19 (953-3100) grams. Refractive errors were the leading type of ocular morbidity seen in 115/222 (51.8%), followed by NLDO (21.2%) (Table 2).

Confidential: For Review Only

Table 2. Types of ocular disorders among premature children screened

Ocular disorders	n	%
Refractive error	115	51.8
Nasolacrimal duct obstruction	47	21.2
Strabismus	25	11.3
Retinopathy of prematurity	16	7.2
Others	5	2.3

NB- Some ocular disorders occur more than once.

Refractive error

One hundred and fifteen (51.8) children had a refractive error, of which 55.5% (81/146) and 44.7% (34/76) of children enrolled from the SPH and TASH had refractive errors, respectively. The mean age at presentation was 2.68 ±0.56 (2.08-6.38) years, and the male-to-female ratio was 1:1.25. Thirty-nine (59%) of children aged > 3 years developed refractive error in comparison with 76 (48.1%) of those aged < 3 years.

The mean gestational age and birthweight of children with refractive errors was 34.11±1.54 (30-36) weeks and 1892.34±414.55 (1080-3100) grams, respectively. Myopia was the commonest type of refractive error, accounting for 78/115 (60.8%) of cases, followed by astigmatism (30, 26.1%) and hyperopia (15, 13.1%). Gender, GA, BW, oxygen supplementation, children, and maternal morbidity were not statistically associated with refractive error. (Table 3)

Strabismus

Twenty-five children (11.3%) had strabismus (5 isolated, 20 in combination with refractive error, nystagmus, ROP, and nasolacrimal duct obstruction). The age at presentation was 2.73±0.52 (2.1-3.6) years, and the male-to-female ratio was 1.08:1.

The mean GA and BW were 34.0±1.41 (30-36) weeks and 906.76±489.92 (1140-3000), respectively. Regarding the types of strabismus, 13 cases had esotropia, and the remaining 12 patients had exotropia. There was no statistically significant association between GA, BW, and strabismus (Table 3). In this study, the prevalence of strabismus among children aged ≥ 3years was 16.7% compared to 8.9% in those < 3 years. However, older age was not statistically associated with strabismus.

Confidential: For Review Only

Retinopathy of prematurity

Previous history of ROP was noted in 16/22 (7.2%) of the children enrolled in this study. Most patients (12, 57%) with ROP were from SPH. Almost all of them (15/16) had a GA < 34 weeks, and the mean GA and BW of patients with ROP were 32.19±1.33 (30-35) weeks and 1596.25 ±483.64 (953-2600) grams, respectively. Nine patients with ROP had an associated refractive error (6 myopia and 3 astigmatism). Only one patient had an associated intermittent exotropia. In univariate analysis, ROP was statistically associated with low GA and low BW (Table 3). Multivariable logistic regression analysis was not conducted due to the small number of children with ROP.

Table 3. Ocular disorders by sex, gestational age, and birthweight among premature children screened for ocular disorders

Type of Disorder	Variables		Yes	No	Odds Ratio	p-Value
			n	n		
**Refractive error**	**Sex**	Male	51	54	0.78(0.46-1.33)	0.361
		Female	64	53		
	**BWt**	<u><</u>1500 gm	22	21	1.03(0.53-2.01)	0.926
		>1500gm	93	86		
	**GA**	<u><</u>34 weeks	62	60	1.09)0.64-1.85)	0.746
		> 34 weeks	53	47		
**Strabismus**	**Sex**	Male	12	93	1.03(0.4501.37)	0.940
		Female	13	104		
	**BWt**	<u><</u>1500 gm	7	36	0.57(0.22-1.48)	0.246
		>1500gm	18	161		
	**GA**	<u><</u>34 weeks	14	108	0.95 (0.41-2.2)	0.911
		> 34 weeks	11	89		
**ROP**	**Sex**	Male	6	99	0.65(0.22-1.85)	0.415
		Female	10	107		
	**BWt**	<u><</u>1500 gm	7	36	Reference	0.01
		>1500gm	9	170	0.27(0.09-0.78)	
	**GA**	<u><</u>34 weeks	15	107	Reference	0.001
		> 34 weeks	1	99	0.72 (0.09-0.56)	

Legend: BWt- Birth Weight ; GA-Gestational Age

Visual impairment and ocular disorders

The mean VA of the right and left eyes was 0.22(SD 0.23) logMAR and 0.17 (SD 0.21) logMAR, respectively. The mean VA in the better and worse eyes was 0.17 (SD 0.22) logMAR

Confidential: For Review Only

and 0.28 (SD 0.21) logMAR, respectively. In this study, 101 (45.9%) and 181 (81.5%) of the children had subnormal visual acuity (>logMAR 0.1) in the better and worst eyes, respectively. Nearly one-fourth (55, 24.8%) of children screened had visual impairment in the better eye. Of this group 51 (92.7%) had uncorrected refractive error alone (34/51) or with strabismus (10/51), ROP (6/51), or nystagmus (1/51). Eighty-nine (40.1%) patients had amblyopia, of which 59/89 (66.3%) had bilateral amblyopia from uncorrected refractive error. Isometropic and anisometropic amblyopia from uncorrected refractive error were the commonest causes of amblyopia, contributing to 49/89 (55%) and 20/89 (22.8%), respectively. Of the 16 cases with ROP, 12 (75%) had a visual impairment associated with other disorders like refractive error, strabismus, and nystagmus.

In univariate analysis, visual impairment in the better eye was statistically associated with ROP, uncorrected refractive error, and strabismus with p-values of 0.001, 0.001, and 0.004, respectively. Amblyopia was not statistically associated with low GA or low BW (Table 4).

Table 4. The presence of visual impairment by types of ocular disorders among premature children screened for ocular disorders.

**Variables**		**Visual Impairment in the better eye**		**Odds Ratio 95% CI (Lower-Upper)**	**p-Value**
		Yes	No		
Sex	Male	23	82	0.75(0.40-1.38)	0.348
	Female	32	85		
**BWt**	≤1500 gm	14	29	0.62(0.29-1.27)	0.188
	>1500gm	41	138		
**GA**	≤34 weeks	28	94	1.24(0.67-2.29)	0.294
	> 34 weeks	27	73		
**Refractive error**	Yes	48	67	10.24(4.37-23.97)	0.001
	No	7	100		
**Strabismus**	Yes	12	13	3.31 (1.41-7.77)	0.004
	No	43	154		

Confidential: For Review Only

DISCUSSION

The present prospective study examines the effects of prematurity on visual acuity and ocular disorder in children born preterm. In Sub-Saharan Africa, neonatal death has decreased by 40% since 1990 due to improved newborn care, likely leading to an increase in childhood ocular morbidity and blindness from diseases like ROP (32). Despite this positive progress, data on the extent of ocular diseases among the preterms in Sub-Saharan Africa are limited. Our study has demonstrated that the prevalence of ocular diseases and visual impairment in Ethiopian children born preterm is high. To our knowledge, this is the first study to assess the prevalence and causes of ocular disorders and visual impairment among children born preterm and admitted to NICUs. In Ethiopia, intensive neonatal care has expanded in many public and private hospital NICUs since 2013 (33), and neonatal mortality per thousand live births has declined modestly from 39 in 2000 to 33 in 2019(34). A prospective screening survey among neonates admitted to two NICUs in Ethiopia showed that 32.2% of the screened infants had any stage ROP (35). However, there is no regular ROP screening program in the country. A comparison of studies of ocular morbidity and visual impairment among preterm children is difficult as there are methodological variations such as differing age groups, inclusion or exclusion of ROP, stages of ROP, and cohort size. Even though genetic and visual experiences predominantly determine the prevalence of refractive error, studies have shown that low BW interrupts emmetropisation and increases the prevalence of refractive error (36). In our study, nearly half of the premature screened children (51.8%) had refractive error, which is comparable to a survey from Turkey (53.8%) (37) but higher than in Italy (42.3%) (38), and in cohorts of extremely preterm infants from Sweden (29.7%) (39) and Norway (10%) (40). In our study, the prevalence of myopia was 35.1%, which was higher than a cohort of preterm children at age 10–12 years from the UK (18.9%) (41), India (15.8%) (42), and Sweden (4.1%) (39). The prevalence of hyperopia in our study, 13.5%, is comparable with that reported in Turkey (14.3%) (37) and Sweden (17.1%) (39) but higher than the UK (6.6%) (41) and India (8.54%) (42). In the present study, 13.5% of the preterm children

Confidential: For Review Only

had clinically significant astigmatism, which was lower than that reported in Norway (21%) (40) and India (55.6%) (42) and higher than in Turkey (5.7%) (37) and Sweden (6.5%) (39). The higher proportion of myopia seen in our study, in comparison with studies from the UK (41), India (42) and Sweden (39), is supported by long-term studies which have confirmed the increased incidence of myopia following preterm birth (43).

Manifest strabismus was seen in 11.3% of our cohort, which is comparable to studies from Norway (10%) (40), the UK (13.6%) (15), Sweden (13.5%) (20), and Australia (14%) (45), and lower than reported in Sweden (17%) (36), the UK and Ireland (24%) *(46)* and Germany (26%) (47). It is unclear at what age the different types of strabismus develop (36), and the age at onset of strabismus in low birthweight children is variable, from the first few months of life to many years later (11, 15, 16,21,22, 44). In our study, a higher prevalence of strabismus in those aged > 3 years was noted. This finding (16.7%) is comparable with a similar age group from Sweden (20). Regarding the type of strabismus, we detected similar proportions for esotropia and exotropia. This is similar to the other studies from Germany (47) and England (41). However, other investigations confirmed that esotropia was the most frequent type of strabismus (20, 39, 48) The increased prevalence of strabismus in the low birthweight population is well-documented (21, 36, and 44). Such an association was not apparent in our study, as most of the children were considerably higher in weight and older than in the studies mentioned above.

The prevalence of ROP in our study is 7.2%, lower than in other studies, from sub-Saharan African countries, including Ethiopia, which ranged from 15-41.7% (35, 49-51). The lower prevalence of ROP in our study can be explained by our data collection method, where we depend on the history of ROP either from the patient's parents or from old features of ROP.

In the present study, 46% of the children had subnormal visual acuity (>logMAR 0.1) in the better eye, which is comparable with a population-based study from Norway (45.9%) (40). The figure is higher than what has been reported for prematurely-born children with BWs 1500–2000 g (15 %) from Denmark (9) and from Sweden 32 % (20). Birch et al. reported significantly lower visual acuities in low birthweight infants compared to those born full term (52). In our study, there was no statistically significant correlation between BCVA and BW or GA, similar to a study from Turkey (37). However, Dowdeswell et al. (53) found low levels of distance visual acuity in preterm children compared with full-term children. However, in our study, ocular

Confidential: For Review Only

morbidities like strabismus, refractive error, and ROP were statistically associated with visual impairment.

In our study, the prevalence of amblyopia among premature children was 40.1%. The result in our study is much higher than other studies from Australia (7.3%) (45) and Turkey (7.7%) (37). Previous studies have shown that prematurity and low birthweight are two risk factors for amblyopia (41, 54). Nevertheless, amblyopia was not statistically associated with low GA and BW. Even if we did not find a statistical association between GA and BW with amblyopia, the prevalence among premature children is higher than in other studies; this indicates that more importance should be given to screening amblyopia risk factors for premature infants.

CONCLUSION

In conclusion, the rates of ocular disorders, visual impairment, and amblyopia in these NICUs in Ethiopia were higher than in other studies. Refractive error, strabismus, and ROP were all significant risk factors for visual impairment. These findings underline the importance of early screening of premature infants for vision and amblyopia. As the two NICUs included in the survey are Ethiopia's main neonatal referral centers, it can be postulated that ocular morbidities, visual impairment, and amblyopia are emerging as potentially avoidable causes of childhood blindness among preterm children in Ethiopia. Developing preterm ocular-related screening protocols within the NICUs, strengthening the referral links between the NICUs and eye centers, and further detailed comparative studies between preterm and term children for ocular disorders are recommended.

The strengths of this study were the prospective controlled study design with a high number of participants, the multi-center design which increases the representativeness of our research, and the availability of medical information from all children and mothers, which allowed a very detailed examination and an adjustment for different possible confounding factors. The strict standardization reduced the probability of examiner-dependent variances.

Confidential: For Review Only

Limitations of the study included the wide age range of the examined children, some of whom were at an early age and phase of refractive development, and other older children that can affect the physiologic refractive changes noted in normal health children. The other limitation is there is a chance that those infants with poor health outcomes did not take part in our study. In subsequent research, we will continue following up with these infants to determine future changes in their refractive error and strabismus.

Lists of Abbreviations

Bronchopulmonary dysplasia (BPD); Necrotizing enterocolitis (NEC); Neonatal Intensive Care Units (NICUs); Patent ductus arteriosus (PDA); Preterm birth (PTBs); Respiratory distress syndrome (RDS); Retinopathy of prematurity (ROP), Saint Paul Hospital (SPH) and Tikur Anbessa Specialized Hospitals (TASH).

Declarations

Ethics Approval and Consent to Participate

The study was conducted following the Helsinki Declaration and after it was approved by the Institute Ethics Committee of Addis Ababa University ((Ref No. 003/2016). All parents or legal guardians provided written informed consent before the examination.

Availability of Data and Materials

All data generated or analysed during this study are included in this published article

Competing Interests

The authors have no conflicts of interest.

Funding

The SIP study was supported by BMG fund.

Authors' Contributions

Drafting of the manuscript: STS., LM., AM. , and AD. Revision of the manuscript for important intellectual content: STS., LM., AM., AD and AA. Conception and design of study: STS., LM., AM. , and AD. Data acquisition, analysis, or interpretation of data: STS., LM., AM., AD., and AA. Approval of final manuscript to be published: STS., LM., AM., AD., and AA. All authors have read and approved the final version of the manuscript.

Confidential: For Review Only

Acknowledgements

The authors wish to acknowledge the assistance of the staff of the SIP project (Ahmed, Beleyu, Efrata, and Wagaye) for their support during data collection. Our special appreciation goes to Sr Martha H/Mariam and Sr. Medhanit from Menelik II pediatrics Ophthalmology clinic.

Confidential: For Review Only

REFERENCES

1. Cao G, Liu J, Liu M. Global, regional, and national incidence and mortality of neonatal preterm birth, 1990-2019. JAMA pediatrics. 2022 Aug 1;176(8):787-96.

2. World Health Organization. 2014. Global Burden of Diseases Estimates http://www.who.int/healthinfo/global_burden_disease/en/.

3. Adane AA, Ayele TA, Ararsa LG, Bitew BD, Zeleke BM. Adverse birth outcomes among deliveries at Gondar University Hospital, Northwest Ethiopia, BMC Pregnancy and Childbirth 2014, 14:90.

4. Hellström A, Smith LE, Dammann O. Retinopathy of prematurity. The lancet. 2013 Oct 26;382(9902):1445-57.

5. Gogate P, Gilbert C, Zin A. Severe visual impairment and blindness in infants: causes and opportunities for control. Middle East Afr J Ophthalmol. 2011;18(2):109–114.

6. Chen C, Zhang QS. [Advances in medical care for extremely low birth weight infants worldwide]. Zhongguo Dang Dai Er Ke Za Zhi. 2013;15(8):703–707. Chinese.

7. Mccolm JR, Fleck BW. Retinopathy of prematurity: causation. Semin Neonatol. 2001;6(6):453–460.

8. Cats BP, Tan KEWP. Prematures with and without regressed retinopathy of prematurity: comparison of long-term (6–10 years) ophthalmological morbidity. J Pediatr Ophthalmol Strab 1989;Nov/Dec:271–5.

9. Fledelius HC, Greisen G. Very pre-term birth and visual impairment. Acta Ophthalmol 1993;Suppl 210:63–5.

10. Gallo JE, Lennerstrand G. A population-based study of ocular abnormalities in premature

children aged 5 to 10 years. Am J Ophthalmol 1991;111:539–47.

11. Holmstrom G, el Azzazi M, Kugelberg U. Ophthalmological follow up of preterm infants: a population based, prospective study of visual acuity and strabismus. Br J Ophthalmol 1999;83:143–50.

12. Sebris SL, Dobson V, Hartmann EE. Assessment and prediction of visual acuity in 3-to 4-year-old children born prior to term. Human Neurobiol 1984;3:87–92.

13. Visual function in low birthweight children A R O'Connor, T J Stephenson, A Johnson, M J Tobin, S Ratib, M Moseley, A R Fielder.

14. Larsson E, Rydberg A, Holmstrom G. Contrast sensitivity in 10 year old preterm and full term children: a population basedstudy. Br J Ophthalmol 2006;90:87–90.

15. Cooke RW, Foulder-Hughes L, Newsham D, Clarke D. Ophthalmic impairment at 7 years of age in children born very preterm. Arch Dis Child Fetal Neonatal Ed 2004;89:F249–53.

16. O'Connor AR, Stephenson TJ, Johnson A, Tobin MJ, Ratib S, Moseley M, et al. Visual function in low birthweight children. Br J Ophthalmol 2004;88:1149–53.

17. Darlow BA, Clemett RS, Horwood LJ, et al. Prospective study of New Zealand infants with birth weight less than 1500 g and screened for retinopathy of prematurity: visual outcome at age 7–8 years. Br J Ophthalmol 1997;81:935–40.

18. Ng YK, Fielder AR, Shaw DE, Levene MI. Epidemiology of retinopathy of prematurity. Lancet 1988; 2(8622): 1235–1238.

19. Holmstrom G, el Azazi M, Jacobson L, Sachs D, Sule J, Lennerstrand G. Epidemiology of ROP in the Stockholm area of Sweden. Acta Opthalmol 1993; 210(Suppl): 44–47.

20. Holmström G, El Azazi M, Kugelberg U. Ophthalmological long term follow up of preterm infants: a population based, prospective study of the refraction and its development. Br J Ophthalmol. 1998;82:1265–1271.

21. Laws D, Shaw DE, Robinson J, et al. Retinopathy of prematurity: a prospective study. Review at six months. Eye 1992;6:477–83.

22. Pennefather PM, Clarke MP, Strong NP, et al. Ocular outcome in children born before 32 weeks gestation. Eye 1995;9(Suppl):26–30.

23. Robinson R, O'Keefe M. Follow-up study on premature infants with and without retinopathy of prematurity. Br J Ophthalmol 1993;77:91–4.

24. McGinnity FG, Bryars JH. Controlled study of ocular morbidity in school children born

Confidential: For Review Only

preterm. Br J Ophthalmol 1992;76:520–4.
25. Asferaw M, Woodruff G, Gilbert C. Causes of severe visual impairment and blindness in students in schools for the blind in Northwest Ethiopia. BMJ Glob Health. 2017;2(2):e000264.
26. Kello AB, Gilbert C. Causes of severe visual impairment and blindness in children in schools for the blind in Ethiopia. Br J Ophthalmol. 2003;87(5):526-30.
27. Muhe, L.M., McClure, E.M., Mekasha, A. et al. A Prospective Study of Causes of Illness and Death in Preterm Infants in Ethiopia: The SIP Study Protocol. Reprod Health 15, 116 (2018). https://doi.org/10.1186/s12978-018-0555-y.
28. Karl S. Connie SN, Wai Suen et al. 2015. Preterm or Not – An Evaluation of Estimates of Gestational Age in a Cohort ofWomen from Rural Papua New Guinea. PLOSONE |DOI:10.1371/journal.pone.0124286 May6, 2015.
29. Rosenberg RE, Nawshad ASM, Ahmed U. et al 2009. Determining Gestational Age in a Low-resource Setting: Validity of Last Menstrual Period. J HEALTH POPUL NUTR 2009 Jun;27(3):332-338.
30. Bach M, Kommerell G. Determining visual acuity using European normal values: scientific principles and possibilities for automatic measurement. Klin Monatsbl Augenheilkd. 1998;212(4):190–5.
31. Hosmer DW, Lemesbow S. Goodness of fit tests for the multiple logistic regression model. Communications in statistics-Theory and Methods. 1980 Jan 1;9(10):1043-69.
32. Hug L, Alexander M, You D, Alkema L. National, regional, and global levels and trends in neonatal mortality between 1990 and 2017, with scenario-based projections to 2030: a systematic analysis. The Lancet Global Health. 2019 Jun 1;7(6):e710-20.
33. UNICEF. Investing in Survival: Enhancing the Neonatal Intensive Care Unit of Yekatit 12 Hospital: A Final Report for UNICEF's Next Generation; 2013 file. https://www.unicefusa.org/sites/default/files/Ethiopia%20Report.pdf. Accessed January 2023.
34. Ministry of Health-Ethiopia. Health Sector Transformation Plan-II. 2021. https://www.globalfinancingfacility.org/ethiopia-health-sector-transformation-plan-201920-202425. Accessed January 2023.
35. Sherief ST, Taye K, Teshome T, Demtse A, Gilbert C. Retinopathy of prematurity among infants admitted to two neonatal intensive care units in Ethiopia. BMJ Open Ophthalmology. 2023 Jul 1;8(1):e001257.

Confidential: For Review Only

36. O'Connor A, Fielder AR. Long term ophthalmic sequelae of prematurity. Early human development. 2008 Feb 1;84(2):101-6.

37. Özdemir M, Koylu S. Ocular growth and morbidity in preterm children without retinopathy of prematurity. Japanese journal of ophthalmology. 2009 Nov;53:623-8.

38. Ricci B. Refractive errors and ocular motility disorders in preterm babies with and without retinopathy of prematurity. Ophthalmologica. 1999 Oct 1;213(5):295-9.

39. Hellgren KM, Tornqvist K, Jakobsson PG, Lundgren P, Carlsson B, Källén K, Serenius F, Hellström A, Holmström G. Ophthalmologic outcome of extremely preterm infants at 6.5 years of age: Extremely Preterm Infants in Sweden Study (EXPRESS). JAMA ophthalmology. 2016 May 1;134(5):555-62.

40. Haugen OH, Nepstad L, Standal OA, Elgen I, Markestad T. Visual function in 6 to 7 year-old children born extremely preterm: a population-based study. Acta ophthalmologica. 2012 Aug;90(5):422-7.

41. O'Connor AR, Stephenson TJ, Johnson A, Tobin MJ, Ratib S, Fielder AR. Change of refractive state and eye size in children of birth weight less than 1701 g. British journal of ophthalmology. 2006 Apr 1;90(4):456-60.

42. Sathar A, Abbas S, Nujum ZT, Benson JL, Sreedevi GP, Saraswathyamma SK. Visual outcome of preterm infants screened in a tertiary care hospital. Middle East African Journal of Ophthalmology. 2019 Jul;26(3):158.

43. O'Connor AR, Wilson CM, Fielder AR. Ophthalmological problems associated with preterm birth. Eye. 2007 Oct;21(10):1254-60.

44. Theng JT, Wong TY, Ling Y. Refractive errors and strabismus in premature Asian infants with and without retinopathy of prematurity. Singap Med J 2000;41:393–7.

45. Hebbandi SB, Bowen JR, Hipwell GC, Ma PJ, Leslie GI, Arnold JD. Ocular sequelae in extremely premature infants at 5 years of age. Journal of paediatrics and child health. 1997 Aug;33(4):339-42.

46. Marlow N,Wolke D, BracewellMA, SamaraM; EPICure Study Group. Neurologic and developmental disability at six years of age after extremely preterm birth. N Engl J Med. 2005;352(1):9-19.

Confidential: For Review Only

47. Fieß A, Kölb-Keerl R, Schuster AK, Knuf M, Kirchhof B, Muether PS, Bauer J. Prevalence and associated factors of strabismus in former preterm and full-term infants between 4 and 10 Years of age. BMC ophthalmology. 2017 Dec;17(1):1-9.

48.VanderVeen DK, Bremer DL, Fellows RR, Hardy RJ, Neely DE, Palmer EA, Rogers DL, Tung B, Good WV, Early Treatment for Retinopathy of Prematurity Cooperative G. Prevalence and course of strabismus through age 6 years in participants of the early treatment for retinopathy of prematurity randomized trial. J AAPOS. 2011;15(6):536–40.

49. Fajolu IB, Rotimi-Samuel A, Aribaba OT, et al. Retinopathy of prematurity and associated factors in Lagos, Nigeria. Paediatr Int Child Health. 2015;35(4):324-8.

50. Omer IM, Hassan HA. The prevalence and risk factors of retinopathy of prematurity among preterm babies admitted to Soba Neonatal Intensive Care Unit. Sudan J Paed. 2014;14(2):17.

51. Wanjala I ID, Kariuki L. Retinopathy of Prematurity as seen in two major hospitals in Nairobi, Kenya. East Afr J Ophthalmol. 2007 May;13(1):5-14.

52. Birch EE, O'Connor AR. Preterm birth and visual development. InSeminars in Neonatology 2001 Dec 1 (Vol. 6, No. 6, pp. 487-497). WB Saunders.

53. Dowdeswell HJ, Slater AM, Broomhall J, Tripp J. Visual defi cits in children born at less than 32 weeks gestation with and without major ocular pathology and cerebral damage. Br J Ophthalmol 1995;79:447–452.

54. Mocanu V, Horhat R. Prevalence and risk factors of amblyopia among refractive errors in an Eastern European population. Medicina. 2018 Mar 20;54(1):6.

Confidential: For Review Only

TABLES LEGEND

Table 1 Characteristics of premature children and mothers screened for ocular disorders

Table 2. Types of ocular disorders among premature children screened

Table 3. Ocular disorders by sex, gestational age, and birthweight among premature children screened for ocular disorders

Table 4. The presence of visual impairment by types of ocular disorders among premature children screened for ocular disorders.

Confidential: For Review Only

BMJ Paediatrics Open

Prevalence and causes of ocular disorders and visual impairment among preterm children in Ethiopia

Journal:	*BMJ Paediatrics Open*
Manuscript ID	bmjpo-2023-002317.R1
Article Type:	Original research
Date Submitted by the Author:	31-Oct-2023
Complete List of Authors:	SHERIEF, SADIK; Addis Ababa University, Department of Ophthalmology; SickKids Research Institute<br>Muhe, Lulu M; Addis Ababa University College of Health Sciences<br>Mekasha, Amha; Addis Ababa University, Department of Pediatrics and Child Health<br>Demtse, Asrat; Addis Ababa University College of Health Sciences, Paediatrics and Child Health<br>Ali, Asim; The Hospital for Sick Children, Ophthalmology and Vision Sciences
Keywords:	Infant, Neonatology, Ophthalmology

SCHOLARONE™
Manuscripts

I, the Submitting Author has the right to grant and does grant on behalf of all authors of the Work (as defined in the below author licence), an exclusive licence and/or a non-exclusive licence for contributions from authors who are: i) UK Crown employees; ii) where BMJ has agreed a CC-BY licence shall apply, and/or iii) in accordance with the terms applicable for US Federal Government officers or employees acting as part of their official duties; on a worldwide, perpetual, irrevocable, royalty-free basis to BMJ Publishing Group Ltd ("BMJ") its licensees and where the relevant Journal is co-owned by BMJ to the co-owners of the Journal, to publish the Work in this journal and any other BMJ products and to exploit all rights, as set out in our licence.

The Submitting Author accepts and understands that any supply made under these terms is made by BMJ to the Submitting Author unless you are acting as an employee on behalf of your employer or a postgraduate student of an affiliated institution which is paying any applicable article publishing charge ("APC") for Open Access articles. Where the Submitting Author wishes to make the Work available on an Open Access basis (and intends to pay the relevant APC), the terms of reuse of such Open Access shall be governed by a Creative Commons licence – details of these licences and which Creative Commons licence will apply to this Work are set out in our licence referred to above.

Other than as permitted in any relevant BMJ Author's Self Archiving Policies, I confirm this Work has not been accepted for publication elsewhere, is not being considered for publication elsewhere and does not duplicate material already published. I confirm all authors consent to publication of this Work and authorise the granting of this licence.

Confidential: For Review Only

Prevalence and causes of ocular disorders and visual impairment among preterm children in Ethiopia

Sadik Taju Sherief [1,2], Lulu Muhe [3] Amha Mekasha [3], Asrat Demtse [3], and Asim Ali[4]

[1] Department of Ophthalmology, Addis Ababa University, Addis Ababa, Ethiopia;

[2] Child Health Evaluative Sciences Program and Centre for Global Child Health, Sickkids Research Institute, Toronto, Canada;

[3] Department of Pediatrics and Child Health, Addis Ababa University, Addis Ababa, Ethiopia;

[4] Department of Ophthalmology and Vision Sciences, The Hospital for Sick Children and University of Toronto, Toronto, Canada.

Short Title: Ocular disorders in Preterms

Corresponding Author:
Sadik T. Sherief, MD
Department of Ophthalmology
School of Medicine, College of Health Sciences
Addis Ababa University
P.O. Box 9086, Addis Ababa, Ethiopia
Tel: +251917801719
Email: goge4000@yahoo.com

Submitted as an Original Research article to: BMJ Paediatrics Open

Abstract word count (300 maximum): 235 words

Manuscript word count (4000 maximum): 3,433 words

Tables and Figures (maximum 5): 4 Tables

Reference Count: 54

Submitted to: *BMJ Paediatrics Open*

Key words: Preterm children; Low birth weight; Visual Impairment; Refractive error; Strabismus; Retinopathy of prematurity; Risk factors of visual impairment; Sub-Saharan Africa; Ethiopia.

Confidential: For Review Only

ABSTRACT

Objective

The aim of this study was to determine the prevalence, causes of ocular disorders and visual impairment among preterm children previously admitted to neonatal intensive care units in Addis Ababa, Ethiopia.

Methods and Analysis

A prospective screening survey was conducted from Feb. to June 2019 at the pediatric eye clinic of Menelik II Hospital. Children who were preterm at birth and who attended the eye clinic were included in the study. Data on demographic and neonatal characteristics, neonatal and maternal co-morbidities, and ocular disorders were collected. Odds ratio and univariate analysis were used to identify predictors of ocular diseases and visual impairment.

Results

There were 222 children included in the study with a mean age at presentation of 2.62 years (range 2.08- 6.38 years), mean GA 34.11 weeks (range 30-36) weeks, and mean birthweight 1941.72g (range 953-3500g). Nearly 2/3 had ocular disorders with refractive error (51.8%), strabismus (11.3%), and history of ROP (7.2%) being more common. One-fourth of the children had visual impairment, and the prevalence of amblyopia was 40.1%. Uncorrected refractive errors, strabismus, and ROP were causes for visual impairment.

Conclusion

Visual impairment and amblyopia are common in Ethiopia. There is a need to develop a screening protocol for ocular disorders for preterm children to enhance early detection and prevention of childhood visual impairment.

Keywords: Preterm children; Low birth weight; Visual Impairment; Refractive error; Strabismus; Retinopathy of prematurity; Risk factors of visual impairment; Sub-Saharan Africa; Ethiopia.

Confidential: For Review Only

Key Messages

What is already known about this subject?

- In many low- and middle-income countries, the survival of preterm infants has improved as neonatal systems have improved.
- Preterm children are at a higher risk of developing ocular disorders, visual impairment, and amblyopia than term children.

What this study adds

- The magnitude and causes of ocular morbidity among preterm children are not well studied in sub-Saharan African countries. This study, conducted among preterm children admitted to two NICUs in a sub-Saharan country, shows that preterm infants develop a higher rate of visual impairment and amblyopia.

How this study might affect research, practice or policy

- The findings of this study provide some evidence for screening for ocular diseases in preterm children, but further studies are needed.

Confidential: For Review Only

INTRODUCTION

Global, regional, and national estimates of preterm birth (defined as childbirth at less than 37 completed weeks) using the 2019 Global Burden of Disease study showed 15.22 million preterm births (1). In the Global Burden of Disease Study, 3.1% of all disability-adjusted life-years were attributed to preterm birth, similar to the burden of HIV or malaria (2). More than 95% of preterm births are occurring in developing countries. Globally the estimated preterm birth rate is 11.1%. Over 60% of preterm births occur in Sub-Saharan Africa and South Asia (1). Ethiopia belongs to the top 15 countries that contribute to two-thirds of the world's preterm babies with a preterm rate of 14.1% out of 481 deliveries (3).

From six months of pregnancy to term is considered the most active period for ocular development (4). Improved neonatal care has increased the survival rates of extremely preterm infants with birth weights (BW) of 1,000 g or gestational age (GA) of 28 weeks; at the same time, retinopathy of prematurity (ROP) has become a significant threat to visual function (5-7). Preterm children are reported to have an increased incidence of visual impairment because of perinatal lesions in the brain (8-10).

It has been noted that both preterm birth and retinopathy of prematurity (ROP) have an effect on the developing visual system, leading to decreased visual acuity, decreased contrast sensitivity, and an increase in color vision deficiencies (11-16). Population-based studies suggest that ophthalmic impairments remain common in very low birth weight infants (11,16,17). Effects of prematurity on ocular and neurological development include retinopathy of prematurity (ROP), refractive error, strabismus, cerebral visual impairment, color vision deficits, reduced contrast sensitivity (CS), visual field defects, and decreased visual acuity (16). According to population studies, the incidence of ROP, whether moderate or severe, for infants born at less than 1500–1700 g ranges from 22–49% (17-19).

In a cohort study, children with lower birth weights had significantly worse near and distance visual acuity at ages 10 to 12 years compared to full-term infants (10). Additionally, infants born prematurely without ROP are more likely to have myopia and anisometropia than infants born at term because preterm babies are more likely to experience refractive errors (20). An increased incidence of strabismus has also been reported in children born prematurely,

Confidential: For Review Only

regardless of the presence of ROP (21-24).

Research on ocular morbidities among preterm infants in sub-Saharan African nations is limited. Before 2020, blindness from ROP was not reported in Ethiopia, including studies in schools for the blind (25, 26).

To determine the top causes of illness and mortality in preterm infants admitted to neonatal intensive units (NICUs) in Ethiopia, an Ethiopian Study of Illness in Preterms (SIP) study was conducted based on standardized diagnostic protocols. This study is part of the SIP study focusing on ocular morbidities among preterms. The present study aimed to identify ocular disorders in a population of preterm children with and without ROP.

METHODS

Study design and subjects

The SIP Study is a prospective study conducted to determine the top causes of illness and mortality in preterm infants admitted to hospitals in Ethiopia based on standardized diagnostic protocols (27). The study participants of this current study are from the SIP study from Feb – June, 2019. The research was performed in accordance with the Declaration of Helsinki and was approved by the Institute Ethics Committee of Addis Ababa University ((Ref No. 003/2016). All parents or legal guardians provided informed consent before the examination. Patients or the public weren’t involved in our research's design, conduct, reporting, or dissemination plans.

Study setting

For the SIP study, standard protocols were developed to undertake a physical examination and laboratory investigation, in particular microbiology, radiologic, and ultrasound examinations. There were initial and follow-up examinations to detect the progress of the preterm infant. Addis Ababa University, Gondar University, Jimma University, and St. Paul Millennium Medical College were included in the SIP study. However, for this ocular morbidity aspect of the SIP study, preterms from Addis Ababa, Tikur Anbessa Hospital, Gandhi Hospital, and St. Paul Millennium Medical College were included in the research.

Recruitment methods

Inclusion criteria were (1) GA < 37 weeks and (2) participation in the SIP study. The preterm children were identified from the SIP database. Parents of all preterm infants received a phone call invitation to participate in our investigation.

Confidential: For Review Only

Assessment of prenatal and postnatal history

History data were assessed from each child's recorded file for the enrolled children. The following data were extracted: antenatal risk factors: maternal age, in vitro fertilization, antenatal corticosteroids, preeclampsia/eclampsia, diabetes, HIV/AIDS, chorioamnionitis, mode of delivery, and multiple births. Neonatal factors included sex, GA, BW, resuscitation in the delivery room, respiratory distress syndrome (RDS), duration of invasive/noninvasive mechanical ventilation and oxygen therapy, intracranial hemorrhage, patent ductus arteriosus (PDA), neonatal sepsis, necrotizing enterocolitis (NEC), number of blood transfusions, and bronchopulmonary dysplasia (BPD). There were no regular ROP screening programmes within the NICUs of the hospitals where the patients were admitted. There was no referral system from the NICUs to Ophthalmology clinic, except if the parents noted a concern. In addition, all parents were interviewed using a standardized protocol to request information concerning medical history of the child and parents, including ocular and general morbidities.

Definitions

Gestational age was determined using last menstrual period [LPM], Ballard and Dubowitz scores and ultrasound assessment. Studies in Papua New Guinea have shown good concordance (0.878, 0.914, and 0.886, respectively) compared to antenatal ultrasound as the gold standard (28). LMP in a low-resource setting such as Bangladesh was found to be a more reliable measure of gestational age than previously thought for the estimation of postnatal gestational age of preterm infants (29).

Preterm infants were further classified as late and moderate preterm (32 to < 37 weeks), very preterm (28 to <32 weeks), and extremely preterm (less than 28 weeks). Glasses were prescribed if there was myopia >1.0D, astigmatism >1.0D, or hypermetropia >+2.0D.

Eye examination

All examinations were performed by the PI and lead author (STS), a pediatric ophthalmologist. Testing of best-corrected visual acuity was performed with Lea symbols until school enrolment, and after that, ETDRS was used in all subjects. In cases of visual acuity below 6/60, depending on the children's age, Lea symbols or Landolt rings were used at a distance of 1 m. Values were converted for analysis into the logarithm of the minimum angle of resolution (logMAR) (30).

Cyclopentolate (0.5%) eye drops were administered three times at 10-min intervals, after which cycloplegic refraction and keratometry were analyzed with an autorefractor (Nidek ARK-1s keratometer, Japan). The spherical equivalent (refractive error) was calculated by adding the spherical value and half of the cylindrical value. Anisometropia was defined as a difference between the patients' eyes of ≥ 1.5 diopters of spherical equivalent. Orthoptic examination for strabismus included the cover-uncover test and alternate cover test, the Hirschberg Test and examination of fixation behavior, as well as the presence or absence of nystagmus after having corrected refractive errors. If a child presented with heterotropia, an alternating prism cover test was added to measure the angle of deviation in prism diopters.

Strabismus was defined as constant or intermittent heterotropia of any dimension at a distance and/or near fixation after correcting refractive error. Classification of strabismus was categorized depending on deviation from the primary position (esotropia or exotropia). An anterior segment examination was done using slit lamp biomicroscopy. A dilated posterior segment examination was conducted using indirect ophthalmoscopy with a 28-diopter lens. Retinopathy of prematurity was diagnosed retrospectively from the patients' chart.

Data analyzed using IBM SPSS 21.0 (SPSS Inc., Chicago, USA). Continuous variables were expressed as the mean ± standard deviation (SD) or as the median when appropriate. Categorical variables were expressed as proportions. The chi-square test was used to analyze the association between categorical variables. Associations between ocular morbidities and continuous and categorical variables were computed using Fisher's exact test and Pearson chi-square (χ^2) test, respectively. Continuous variables were compared using ANOVA. Values of $p < 0.05$ were considered statistically significant.

RESULTS

During the study period 222 infants (146 from Saint Paul Hospital and 76 from TASH) were included in this study.

Characteristics of the study population

Slightly more females than males were screened (52.7% and 47.3%, respectively). The majority of the study participants (n=156, 70.3%) were less than 3 years of age and the mean age at presentation was 2.62 ± 0.49 years (range 2.08- 6.38). One hundred and twenty-three of the 222 children (55.4%) had a GA ≤34 weeks and 43 (19.4%) had a BW ≤1500g. Birthweight ranged from 953-3500g with a mean of 1941.72g (SD 445.49); GA ranged from 30-36 weeks, with a mean of 34.11 weeks (SD 1.47). One hundred and twenty-three children (55.4%) were delivered vaginally, and 80 (36.1%) had multiple gestations. Forty-eight children (21.7%) were born to mothers with pregnancy-induced hypertension, and eight (3.7%) mothers tested positive for HIV (Table 1).

The mean BWs of children from SPH and TASH NICUs were 1888.5 ± 403.6 (953-3000) g and 2043.94 ± 503.74 (1125-3500) g, respectively; mean GAs were 34.14 ± 1.49 (30-36) weeks and 34.08 ± 1.44 (30-36) weeks, respectively. Differences in these parameters were not statistically significant (Table 2).

Confidential: For Review Only

Table 1 Characteristics of premature children and mothers screened for ocular disorders

Variable		Total	Male		Female	
		N	N	%	N	%
Birthweight	≤1500 gm	43	20	46.5%	23	53.5%
	>1500gm	179	85	47.5%	94	52.5%
Gestational age	≤34 weeks	122	60	49.2%	62	50.8%
	> 34 weeks	100	45	45%	55	55%
Multiple gestation	Yes	80	42	52.5%	38	47.5%
	No	142	63	44.4%	79	55.6%
Oxygen supplementation	Yes	97	47	48.5%	50	51.5%
	No	125	58	46.4%	67	53.6%
Infantile morbidity	Sepsis	6	2	33.3%	4	66.7%
	IVH	2	0	0%	2	100%
	BPD and Sepsis	1	1	100%	0	0%
	None	213	102	47.9%	111	52.1%
Mode of delivery	Vaginal delivery	123	53	43.1%	70	56.9%
	Cesarean section	99	52	52.5%	47	47.5%
Multiparity	Yes	47	18	38.3%	29	61.7%
	No	175	63	34.3	59	65.7%
Maternal morbidity	PIH	44	22	50%	22	50%
	HIV	5	2	40%	3	60%
	HIV & PIH	3	3	100%	0	0%
	DM	2	0	0%	2	100%
	DM & PIH	1	0	0%	1	100%
	TORCH	1	1	100%	0	0%
	None	166	77	46.4%	89	53.6%
NICU location	SPH	146	65	44.5%	81	55.5%
	TASH	76	40	52,6%	36	47.4%

Legend: BPD- Bronchopulmonary Dysplasia; DM- Diabetes Mellitus; HIV-IVH-Intraventricular Hemorrhage; PIH- Pregnancy-Induced Hypertension; SPH- Saint Paul Hospital; TASH-Tikur Anbessa Specialized Hospital; TORCH- Toxoplasmosis, Rubella, Cytomegalovirus, Herpes simplex, and HIV

Ocular morbidities and risk factors

Overall, 145 (65.3%) of the children had ocular disorders at the presentation, of which 92 (63.4%) had isolated ocular diseases (69 refractive error, 13 nasolacrimal duct obstruction, five strabismus, and five ROP). The mean age at presentation of children with ocular disorders was 2.7±0.5 (2.1- 6.4) years, and there were more females with a male to female ratio of 1:1.27. None of the eyes examined had anomalies of the anterior segment or lens.

The mean GA was 34.14 ± 1.49 (30-36) weeks, and BW was 1927.27 ± 429.19 (953-3100) grams. Refractive errors were the leading type of ocular morbidity seen in 115/222 (51.8%), followed by NLDO (21.2%) (Table 2).

Confidential: For Review Only

Table 2. Types of ocular disorders among premature children screened

Ocular disorders	n	%
Refractive error	115	51.8
Nasolacrimal duct obstruction	47	21.2
Strabismus	25	11.3
Retinopathy of prematurity	16	7.2
Others	5	2.3

NB- Some ocular disorders occur more than once.

Refractive error

One hundred and fifteen (51.8) children had a refractive error, of which 55.5% (81/146) and 44.7% (34/76) of children enrolled from the SPH and TASH had refractive errors, respectively. The mean age at presentation was 2.68 ±0.56 (2.08-6.38) years, and the male-to-female ratio was 1:1.25. Thirty-nine (59%) of children aged > 3 years developed refractive error in comparison with 76 (48.1%) of those aged < 3 years.

The mean gestational age and birthweight of children with refractive errors was 34.11±1.54 (30-36) weeks and 1892.34±414.55 (1080-3100) grams, respectively. Myopia was the commonest type of refractive error, accounting for 78/115 (60.8%) of cases, followed by astigmatism (30, 26.1%) and hyperopia (15, 13.1%). Gender, GA, BW, oxygen supplementation, children, and maternal morbidity were not statistically associated with refractive error. (Table 3)

Strabismus

Twenty-five children (11.3%) had strabismus (5 isolated, 20 in combination with refractive error, nystagmus, ROP, and nasolacrimal duct obstruction). The age at presentation was 2.73±0.52 (2.1-3.6) years, and the male-to-female ratio was 1.08:1.

The mean GA and BW were 34.0±1.41 (30-36) weeks and 906.76±489.92 (1140-3000), respectively. Regarding the types of strabismus, 13 cases had esotropia, and the remaining 12 patients had exotropia. There was no statistically significant association between GA, BW, and strabismus (Table 3). In this study, the prevalence of strabismus among children aged ≥ 3years was 16.7% compared to 8.9% in those < 3 years. However, older age was not statistically associated with strabismus.

Confidential: For Review Only

Retinopathy of prematurity

Previous history of ROP was noted in 16/22 (7.2%) of the children enrolled in this study. Most patients (12, 57%) with ROP were from SPH. Almost all of them (15/16) had a GA < 34 weeks, and the mean GA and BW of patients with ROP were 32.19±1.33 (30-35) weeks and 1596.25 ±483.64 (953-2600) grams, respectively. Nine patients with ROP had an associated refractive error (6 myopia and 3 astigmatism). Only one patient had an associated intermittent exotropia. In univariate analysis, ROP was statistically associated with low GA and low BW (Table 3). Multivariable logistic regression analysis was not conducted due to the small number of children with ROP.

Table 3. Ocular disorders by sex, gestational age, and birthweight among premature children screened for ocular disorders

Type of Disorder	Variables		Yes	No	Odds Ratio	p-Value
			n	n		
Refractive error	Sex	Male	51	54	0.78(0.46-1.33)	0.361
		Female	64	53		
	BWt	≤1500 gm	22	21	1.03(0.53-2.01)	0.926
		>1500gm	93	86		
	GA	≤34 weeks	62	60	1.09)0.64-1.85)	0.746
		> 34 weeks	53	47		
Strabismus	Sex	Male	12	93	1.03(0.4501.37)	0.940
		Female	13	104		
	BWt	≤1500 gm	7	36	0.57(0.22-1.48)	0.246
		>1500gm	18	161		
	GA	≤34 weeks	14	108	0.95 (0.41-2.2)	0.911
		> 34 weeks	11	89		
ROP	Sex	Male	6	99	0.65(0.22-1.85)	0.415
		Female	10	107		
	BWt	≤1500 gm	7	36	Reference	0.01
		>1500gm	9	170	0.27(0.09-0.78)	
	GA	≤34 weeks	15	107	Reference	0.001
		> 34 weeks	1	99	0.72 (0.09-0.56)	

Legend: BWt- Birth Weight ; GA-Gestational Age

Visual impairment and ocular disorders

The mean VA of the right and left eyes was 0.22(SD 0.23) logMAR and 0.17 (SD 0.21) logMAR, respectively. The mean VA in the better and worse eyes was 0.17 (SD 0.22) logMAR and 0.28 (SD 0.21) logMAR, respectively. In this study, 101 (45.9%) and 181 (81.5%) of the children had subnormal visual acuity (>logMAR 0.1) in the better and worst eyes, respectively.

Confidential: For Review Only

Nearly one-fourth (55, 24.8%) of children screened had visual impairment in the better eye. Of this group 51 (92.7%) had uncorrected refractive error alone (34/51) or with strabismus (10/51), ROP (6/51), or nystagmus (1/51). Eighty-nine (40.1%) patients had amblyopia, of which 59/89 (66.3%) had bilateral amblyopia from uncorrected refractive error. Isometropic and anisometropic amblyopia from uncorrected refractive error were the commonest causes of amblyopia, contributing to 49/89 (55%) and 20/89 (22.8%), respectively. Of the 16 cases with ROP, 12 (75%) had a visual impairment associated with other disorders like refractive error, strabismus, and nystagmus.

In univariate analysis, visual impairment in the better eye was statistically associated with ROP, uncorrected refractive error, and strabismus with p-values of 0.001, 0.001, and 0.004, respectively. Amblyopia was not statistically associated with low GA or low BW (Table 4).

Table 4. The presence of visual impairment by types of ocular disorders among premature children screened for ocular disorders.

**Variables**		**Visual Impairment in the better eye**		**Odds Ratio 95% CI (Lower-Upper)**	**p-Value**
		Yes	No		
Sex	Male	23	82	0.75(0.40-1.38)	0.348
	Female	32	85		
**BWt**	≤1500 gm	14	29	0.62(0.29-1.27)	0.188
	>1500gm	41	138		
**GA**	≤34 weeks	28	94	1.24(0.67-2.29)	0.294
	> 34 weeks	27	73		
**Refractive error**	Yes	48	67	10.24(4.37-23.97)	0.001
	No	7	100		
**Strabismus**	Yes	12	13	3.31 (1.41-7.77)	0.004
	No	43	154		

Confidential: For Review Only

DISCUSSION

The present prospective study examines the effects of prematurity on visual acuity and ocular disorder in children born preterm. In Sub-Saharan Africa, neonatal death has decreased by 40% since 1990 due to improved newborn care, likely leading to an increase in childhood ocular morbidity and blindness from diseases like ROP (32). Despite this positive progress, data on the extent of ocular diseases among the preterms in Sub-Saharan Africa are limited. Our study has demonstrated that the prevalence of ocular diseases and visual impairment in Ethiopian children born preterm is high. To our knowledge, this is the first study to assess the prevalence and causes of ocular disorders and visual impairment among children born preterm and admitted to NICUs. In Ethiopia, intensive neonatal care has expanded in many public and private hospital NICUs since 2013 (33), and neonatal mortality per thousand live births has declined modestly from 39 in 2000 to 33 in 2019(34). A prospective screening survey among neonates admitted to two NICUs in Ethiopia showed that 32.2% of the screened infants had any stage ROP (35). However, there is no regular ROP screening program in the country. A comparison of studies of ocular morbidity and visual impairment among preterm children is difficult as there are methodological variations such as differing age groups, inclusion or exclusion of ROP, stages of ROP, and cohort size. Even though genetic and visual experiences predominantly determine the prevalence of refractive error, studies have shown that low BW interrupts emmetropisation and increases the prevalence of refractive error (36). In our study, nearly half of the premature screened children (51.8%) had refractive error, which is comparable to a survey from Turkey (53.8%) (37) but higher than in Italy (42.3%) (38), and in cohorts of extremely preterm infants from Sweden (29.7%) (39) and Norway (10%) (40). In our study, the prevalence of myopia was 35.1%, which was higher than a cohort of preterm children at age 10–12 years from the UK (18.9%) (41), India (15.8%) (42), and Sweden (4.1%) (39). The prevalence of hyperopia in our study, 13.5%, is comparable with that reported in Turkey (14.3%) (37) and Sweden (17.1%) (39) but higher than the UK (6.6%) (41) and India (8.54%) (42). In the present study, 13.5% of the preterm children had clinically significant astigmatism, which was lower than that reported in Norway (21%) (40) and India (55.6%) (42) and higher than in Turkey (5.7%) (37) and Sweden (6.5%) (39). The higher proportion of myopia seen in our study, in comparison with studies from the UK (41),

Confidential: For Review Only

India (42) and Sweden (39), is supported by long-term studies which have confirmed the increased incidence of myopia following preterm birth (43).

Manifest strabismus was seen in 11.3% of our cohort, which is comparable to studies from Norway (10%) (40), the UK (13.6%) (15), Sweden (13.5%) (20), and Australia (14%) (45), and lower than reported in Sweden (17%) (36), the UK and Ireland (24%) *(46)* and Germany (26%) (47). It is unclear at what age the different types of strabismus develop (36), and the age at onset of strabismus in low birthweight children is variable, from the first few months of life to many years later (11, 15, 16,21,22, 44). In our study, a higher prevalence of strabismus in those aged > 3 years was noted. This finding (16.7%) is comparable with a similar age group from Sweden (20). Regarding the type of strabismus, we detected similar proportions for esotropia and exotropia. This is similar to the other studies from Germany (47) and England (41). However, other investigations confirmed that esotropia was the most frequent type of strabismus (20, 39, 48) The increased prevalence of strabismus in the low birthweight population is well-documented (21, 36, and 44). Such an association was not apparent in our study, as most of the children were considerably higher in weight and older than in the studies mentioned above.

The prevalence of ROP in our study is 7.2%, lower than in other studies, from sub-Saharan African countries, including Ethiopia, which ranged from 15-41.7% (35, 49-51). The lower prevalence of ROP in our study can be explained by our data collection method, where we depend on the history of ROP either from the patient's parents or from old features of ROP.

In the present study, 46% of the children had subnormal visual acuity (>logMAR 0.1) in the better eye, which is comparable with a population-based study from Norway (45.9%) (40). The figure is higher than what has been reported for prematurely-born children with BWs 1500–2000 g (15 %) from Denmark (9) and from Sweden 32 % (20). Birch et al. reported significantly lower visual acuities in low birthweight infants compared to those born full term (52). In our study, there was no statistically significant correlation between BCVA and BW or GA, similar to a study from Turkey (37). However, Dowdeswell et al. (53) found low levels of distance visual acuity in preterm children compared with full-term children. However, in our study, ocular morbidities like strabismus, refractive error, and ROP were statistically associated with visual impairment.

In our study, the prevalence of amblyopia among premature children was 40.1%. The result in our study is much higher than other studies from Australia (7.3%) (45) and Turkey

Confidential: For Review Only

(7.7%) (37). Previous studies have shown that prematurity and low birthweight are two risk factors for amblyopia (41, 54). Nevertheless, amblyopia was not statistically associated with low GA and BW. Even if we did not find a statistical association between GA and BW with amblyopia, the prevalence among premature children is higher than in other studies; this indicates that more importance should be given to screening amblyopia risk factors for premature infants.

CONCLUSION

In conclusion, the rates of ocular disorders, visual impairment, and amblyopia in these NICUs in Ethiopia were higher than in other studies. Refractive error, strabismus, and ROP were all significant risk factors for visual impairment. These findings underline the importance of early screening of premature infants for vision and amblyopia. As the two NICUs included in the survey are Ethiopia's main neonatal referral centers, it can be postulated that ocular morbidities, visual impairment, and amblyopia are emerging as potentially avoidable causes of childhood blindness among preterm children in Ethiopia. Developing preterm ocular-related screening protocols within the NICUs, strengthening the referral links between the NICUs and eye centers, and further detailed comparative studies between preterm and term children for ocular disorders are recommended.

The strengths of this study were the prospective controlled study design with a high number of participants, the multi-center design which increases the representativeness of our research, and the availability of medical information from all children and mothers, which allowed a very detailed examination and an adjustment for different possible confounding factors. The strict standardization reduced the probability of examiner-dependent variances.

Limitations of the study included the wide age range of the examined children, some of whom were at an early age and phase of refractive development, and other older children that can affect the physiologic refractive changes noted in normal health children. The other limitation is there is a chance that those infants with poor health outcomes did not take part in our study. In subsequent research, we will continue following up with these infants to determine future changes in their refractive error and strabismus.

Confidential: For Review Only

Lists of Abbreviations

Bronchopulmonary dysplasia (BPD); Necrotizing enterocolitis (NEC); Neonatal Intensive Care Units (NICUs); Patent ductus arteriosus (PDA); Preterm birth (PTBs); Respiratory distress syndrome (RDS); Retinopathy of prematurity (ROP), Saint Paul Hospital (SPH) and Tikur Anbessa Specialized Hospitals (TASH).

Research Ethics Approval

This study involves human participants, and this research was approved by the ethics committee of Addis Ababa University Ethics Review Committee (Ref No. 003/2016) in line with the relevant national and institutional guidelines on care and clinical research. All parents or legal guardians gave written informed consent before participating in the study.

Availability of Data and Materials

All data generated or analysed during this study are included in this published article

Competing Interests

The authors have no conflicts of interest.

Funding

The SIP study was supported by the Bill & Melinda Gates Foundation (OPP1136965)

Authors' Contributions

Drafting of the manuscript: STS., LM., AM. , and AD. Revision of the manuscript for important intellectual content: STS., LM., AM., AD and AA. Conception and design of study: STS., LM., AM. , and AD. Data acquisition, analysis, or interpretation of data: STS., LM., AM., AD., and AA. Approval of final manuscript to be published: STS., LM., AM., AD., and AA. All authors have read and approved the final version of the manuscript.

Acknowledgements

The authors wish to acknowledge the assistance of the staff of the SIP project (Ahmed, Beleyu, Efrata, and Wagaye) for their support during data collection. Our special appreciation goes to Sr Martha H/Mariam and Sr. Medhanit from Menelik II pediatrics Ophthalmology clinic.

Confidential: For Review Only

REFERENCES

1. Cao G, Liu J, Liu M. Global, regional, and national incidence and mortality of neonatal preterm birth, 1990-2019. JAMA pediatrics. 2022 Aug 1;176(8):787-96.

2. World Health Organization. 2014. Global Burden of Diseases Estimates http://www.who.int/healthinfo/global_burden_disease/en/.

3. Adane AA, Ayele TA, Ararsa LG, Bitew BD, Zeleke BM. Adverse birth outcomes among deliveries at Gondar University Hospital, Northwest Ethiopia, BMC Pregnancy and Childbirth 2014, 14:90.

4. Hellström A, Smith LE, Dammann O. Retinopathy of prematurity. The lancet. 2013 Oct 26;382(9902):1445-57.

5. Gogate P, Gilbert C, Zin A. Severe visual impairment and blindness in infants: causes and opportunities for control. Middle East Afr J Ophthalmol. 2011;18(2):109–114.

6. Chen C, Zhang QS. [Advances in medical care for extremely low birth weight infants worldwide]. Zhongguo Dang Dai Er Ke Za Zhi. 2013;15(8):703–707. Chinese.

7. Mccolm JR, Fleck BW. Retinopathy of prematurity: causation. Semin Neonatol. 2001;6(6):453–460.

8. Cats BP, Tan KEWP. Prematures with and without regressed retinopathy of prematurity: comparison of long-term (6–10 years) ophthalmological morbidity. J Pediatr Ophthalmol Strab 1989;Nov/Dec:271–5.

9. Fledelius HC, Greisen G. Very pre-term birth and visual impairment. Acta Ophthalmol 1993;Suppl 210:63–5.

10. Gallo JE, Lennerstrand G. A population-based study of ocular abnormalities in premature children aged 5 to 10 years. Am J Ophthalmol 1991;111:539–47.

11. Holmstrom G, el Azzazi M, Kugelberg U. Ophthalmological follow up of preterm infants: a population based, prospective study of visual acuity and strabismus. Br J Ophthalmol 1999;83:143–50.

12. Sebris SL, Dobson V, Hartmann EE. Assessment and prediction of visual acuity in 3-to 4-year-old children born prior to term. Human Neurobiol 1984;3:87–92.

13. Visual function in low birthweight children A R O'Connor, T J Stephenson, A Johnson, M J Tobin, S Ratib, M Moseley, A R Fielder.

Confidential: For Review Only

14. Larsson E, Rydberg A, Holmstrom G. Contrast sensitivity in 10 year old preterm and full term children: a population basedstudy. Br J Ophthalmol 2006;90:87–90.

15. Cooke RW, Foulder-Hughes L, Newsham D, Clarke D. Ophthalmic impairment at 7 years of age in children born very preterm. Arch Dis Child Fetal Neonatal Ed 2004;89:F249–53.

16. O'Connor AR, Stephenson TJ, Johnson A, Tobin MJ, Ratib S, Moseley M, et al. Visual function in low birthweight children. Br J Ophthalmol 2004;88:1149–53.

17. Darlow BA, Clemett RS, Horwood LJ, et al. Prospective study of New Zealand infants with birth weight less than 1500 g and screened for retinopathy of prematurity: visual outcome at age 7–8 years. Br J Ophthalmol 1997;81:935–40.

18. Ng YK, Fielder AR, Shaw DE, Levene MI. Epidemiology of retinopathy of prematurity. Lancet 1988; 2(8622): 1235–1238.

19. Holmstrom G, el Azazi M, Jacobson L, Sachs D, Sule J, Lennerstrand G. Epidemiology of ROP in the Stockholm area of Sweden. Acta Opthalmol 1993; 210(Suppl): 44–47.

20. Holmström G, El Azazi M, Kugelberg U. Ophthalmological long term follow up of preterm infants: a population based, prospective study of the refraction and its development. Br J Ophthalmol. 1998;82:1265–1271.

21. Laws D, Shaw DE, Robinson J, et al. Retinopathy of prematurity: a prospective study. Review at six months. Eye 1992;6:477–83.

22. Pennefather PM, Clarke MP, Strong NP, et al. Ocular outcome in children born before 32 weeks gestation. Eye 1995;9(Suppl):26–30.

23. Robinson R, O'Keefe M. Follow-up study on premature infants with and without retinopathy of prematurity. Br J Ophthalmol 1993;77:91–4.

24. McGinnity FG, Bryars JH. Controlled study of ocular morbidity in school children born preterm. Br J Ophthalmol 1992;76:520–4.

25. Asferaw M, Woodruff G, Gilbert C. Causes of severe visual impairment and blindness in students in schools for the blind in Northwest Ethiopia. BMJ Glob Health. 2017;2(2):e000264.

26. Kello AB, Gilbert C. Causes of severe visual impairment and blindness in children in schools for the blind in Ethiopia. Br J Ophthalmol. 2003;87(5):526-30.

27. Muhe, L.M., McClure, E.M., Mekasha, A. et al. A Prospective Study of Causes of Illness and Death in Preterm Infants in Ethiopia: The SIP Study Protocol. Reprod Health 15, 116 (2018). https://doi.org/10.1186/s12978-018-0555-y.

Confidential: For Review Only

28. Karl S. Connie SN, Wai Suen et al. 2015. Preterm or Not – An Evaluation of Estimates of Gestational Age in a Cohort ofWomen from Rural Papua New Guinea. PLOSONE |DOI:10.1371/journal.pone.0124286 May6, 2015.

29. Rosenberg RE, Nawshad ASM, Ahmed U. et al 2009. Determining Gestational Age in a Low-resource Setting: Validity of Last Menstrual Period. J HEALTH POPUL NUTR 2009 Jun;27(3):332-338.

30. Bach M, Kommerell G. Determining visual acuity using European normal values: scientific principles and possibilities for automatic measurement. Klin Monatsbl Augenheilkd. 1998;212(4):190–5.

31. Hosmer DW, Lemesbow S. Goodness of fit tests for the multiple logistic regression model. Communications in statistics-Theory and Methods. 1980 Jan 1;9(10):1043-69.

32. Hug L, Alexander M, You D, Alkema L. National, regional, and global levels and trends in neonatal mortality between 1990 and 2017, with scenario-based projections to 2030: a systematic analysis. The Lancet Global Health. 2019 Jun 1;7(6):e710-20.

33. UNICEF. Investing in Survival: Enhancing the Neonatal Intensive Care Unit of Yekatit 12 Hospital: A Final Report for UNICEF's Next Generation; 2013 file. https://www.unicefusa.org/sites/default/files/Ethiopia%20Report.pdf. Accessed January 2023.

34. Ministry of Health-Ethiopia. Health Sector Transformation Plan-II. 2021. https://www.globalfinancingfacility.org/ethiopia-health-sector-transformation-plan-201920-202425. Accessed January 2023.

35. Sherief ST, Taye K, Teshome T, Demtse A, Gilbert C. Retinopathy of prematurity among infants admitted to two neonatal intensive care units in Ethiopia. BMJ Open Ophthalmology. 2023 Jul 1;8(1):e001257.

36. O'Connor A, Fielder AR. Long term ophthalmic sequelae of prematurity. Early human development. 2008 Feb 1;84(2):101-6.

37. Özdemir M, Koylu S. Ocular growth and morbidity in preterm children without retinopathy of prematurity. Japanese journal of ophthalmology. 2009 Nov;53:623-8.

38. Ricci B. Refractive errors and ocular motility disorders in preterm babies with and without retinopathy of prematurity. Ophthalmologica. 1999 Oct 1;213(5):295-9.

39. Hellgren KM, Tornqvist K, Jakobsson PG, Lundgren P, Carlsson B, Källén K, Serenius F, Hellström A, Holmström G. Ophthalmologic outcome of extremely preterm infants at 6.5 years

Confidential: For Review Only

of age: Extremely Preterm Infants in Sweden Study (EXPRESS). JAMA ophthalmology. 2016 May 1;134(5):555-62.

40. Haugen OH, Nepstad L, Standal OA, Elgen I, Markestad T. Visual function in 6 to 7 year-old children born extremely preterm: a population-based study. Acta ophthalmologica. 2012 Aug;90(5):422-7.

41. O'Connor AR, Stephenson TJ, Johnson A, Tobin MJ, Ratib S, Fielder AR. Change of refractive state and eye size in children of birth weight less than 1701 g. British journal of ophthalmology. 2006 Apr 1;90(4):456-60.

42. Sathar A, Abbas S, Nujum ZT, Benson JL, Sreedevi GP, Saraswathyamma SK. Visual outcome of preterm infants screened in a tertiary care hospital. Middle East African Journal of Ophthalmology. 2019 Jul;26(3):158.

43. O'Connor AR, Wilson CM, Fielder AR. Ophthalmological problems associated with preterm birth. Eye. 2007 Oct;21(10):1254-60.

44. Theng JT, Wong TY, Ling Y. Refractive errors and strabismus in premature Asian infants with and without retinopathy of prematurity. Singap Med J 2000;41:393–7.

45. Hebbandi SB, Bowen JR, Hipwell GC, Ma PJ, Leslie GI, Arnold JD. Ocular sequelae in extremely premature infants at 5 years of age. Journal of paediatrics and child health. 1997 Aug;33(4):339-42.

46. Marlow N,Wolke D, BracewellMA, SamaraM; EPICure Study Group. Neurologic and developmental disability at six years of age after extremely preterm birth. N Engl J Med. 2005;352(1):9-19.

47. Fieß A, Kölb-Keerl R, Schuster AK, Knuf M, Kirchhof B, Muether PS, Bauer J. Prevalence and associated factors of strabismus in former preterm and full-term infants between 4 and 10 Years of age. BMC ophthalmology. 2017 Dec;17(1):1-9.

48.VanderVeen DK, Bremer DL, Fellows RR, Hardy RJ, Neely DE, Palmer EA, Rogers DL, Tung B, Good WV, Early Treatment for Retinopathy of Prematurity Cooperative G. Prevalence and course of strabismus through age 6 years in participants of the early treatment for retinopathy of prematurity randomized trial. J AAPOS. 2011;15(6):536–40.

49. Fajolu IB, Rotimi-Samuel A, Aribaba OT, et al. Retinopathy of prematurity and associated factors in Lagos, Nigeria. Paediatr Int Child Health. 2015;35(4):324-8.

Confidential: For Review Only

50. Omer IM, Hassan HA. The prevalence and risk factors of retinopathy of prematurity among preterm babies admitted to Soba Neonatal Intensive Care Unit. Sudan J Paed. 2014;14(2):17.
51. Wanjala I ID, Kariuki L. Retinopathy of Prematurity as seen in two major hospitals in Nairobi, Kenya. East Afr J Ophthalmol. 2007 May;13(1):5-14.
52. Birch EE, O'Connor AR. Preterm birth and visual development. InSeminars in Neonatology 2001 Dec 1 (Vol. 6, No. 6, pp. 487-497). WB Saunders.
53. Dowdeswell HJ, Slater AM, Broomhall J, Tripp J. Visual defi cits in children born at less than 32 weeks gestation with and without major ocular pathology and cerebral damage. Br J Ophthalmol 1995;79:447–452.
54. Mocanu V, Horhat R. Prevalence and risk factors of amblyopia among refractive errors in an Eastern European population. Medicina. 2018 Mar 20;54(1):6.

Confidential: For Review Only

TABLES LEGEND

Table 1 Characteristics of premature children and mothers screened for ocular disorders

Table 2. Types of ocular disorders among premature children screened

Table 3. Ocular disorders by sex, gestational age, and birthweight among premature children screened for ocular disorders

Table 4. The presence of visual impairment by types of ocular disorders among premature children screened for ocular disorders.

Confidential: For Review Only

BMJ Paediatrics Open

Prevalence and causes of ocular disorders and visual impairment among preterm children in Ethiopia

Journal:	*BMJ Paediatrics Open*
Manuscript ID	bmjpo-2023-002317.R2
Article Type:	Original research
Date Submitted by the Author:	05-Jan-2024
Complete List of Authors:	SHERIEF, SADIK; Addis Ababa University, Department of Ophthalmology; SickKids Research Institute<br>Muhe, Lulu M; Addis Ababa University College of Health Sciences<br>Mekasha, Amha; Addis Ababa University, Department of Pediatrics and Child Health<br>Demtse, Asrat; Addis Ababa University College of Health Sciences, Paediatrics and Child Health<br>Ali, Asim; The Hospital for Sick Children, Ophthalmology and Vision Sciences
Keywords:	Infant, Neonatology, Ophthalmology

SCHOLARONE™
Manuscripts

I, the Submitting Author has the right to grant and does grant on behalf of all authors of the Work (as defined in the below author licence), an exclusive licence and/or a non-exclusive licence for contributions from authors who are: i) UK Crown employees; ii) where BMJ has agreed a CC-BY licence shall apply, and/or iii) in accordance with the terms applicable for US Federal Government officers or employees acting as part of their official duties; on a worldwide, perpetual, irrevocable, royalty-free basis to BMJ Publishing Group Ltd ("BMJ") its licensees and where the relevant Journal is co-owned by BMJ to the co-owners of the Journal, to publish the Work in this journal and any other BMJ products and to exploit all rights, as set out in our licence.

The Submitting Author accepts and understands that any supply made under these terms is made by BMJ to the Submitting Author unless you are acting as an employee on behalf of your employer or a postgraduate student of an affiliated institution which is paying any applicable article publishing charge ("APC") for Open Access articles. Where the Submitting Author wishes to make the Work available on an Open Access basis (and intends to pay the relevant APC), the terms of reuse of such Open Access shall be governed by a Creative Commons licence – details of these licences and which Creative Commons licence will apply to this Work are set out in our licence referred to above.

Other than as permitted in any relevant BMJ Author's Self Archiving Policies, I confirm this Work has not been accepted for publication elsewhere, is not being considered for publication elsewhere and does not duplicate material already published. I confirm all authors consent to publication of this Work and authorise the granting of this licence.

Confidential: For Review Only

Prevalence and causes of ocular disorders and visual impairment among preterm children in Ethiopia

Sadik Taju Sherief [1,2], Lulu Muhe [3] Amha Mekasha [3], Asrat Demtse [3], and Asim Ali[4]

[1] Department of Ophthalmology, Addis Ababa University, Addis Ababa, Ethiopia;

[2] Child Health Evaluative Sciences Program and Centre for Global Child Health, Sickkids Research Institute, Toronto, Canada;

[3] Department of Pediatrics and Child Health, Addis Ababa University, Addis Ababa, Ethiopia;
[4] Department of Ophthalmology and Vision Sciences, The Hospital for Sick Children and University of Toronto, Toronto, Canada.

Short Title: Ocular disorders in Preterms

Corresponding Author:
Sadik T. Sherief, MD
Department of Ophthalmology
School of Medicine, College of Health Sciences
Addis Ababa University
P.O. Box 9086, Addis Ababa, Ethiopia
Tel: +251917801719
Email: goge4000@yahoo.com

Submitted as an Original Research article to: BMJ Paediatrics Open

Abstract word count (300 maximum): 235 words

Manuscript word count (4000 maximum): 3,433 words

Tables and Figures (maximum 5): 4 Tables

Reference Count: 54

Submitted to: *BMJ Paediatrics Open*

Key words: Preterm children; Low birth weight; Visual Impairment; Refractive error; Strabismus; Retinopathy of prematurity; Risk factors of visual impairment; Sub-Saharan Africa; Ethiopia.

Confidential: For Review Only

ABSTRACT

Objective

The aim of this study was to determine the prevalence, causes of ocular disorders and visual impairment among preterm children previously admitted to neonatal intensive care units in Addis Ababa, Ethiopia.

Methods and Analysis

A prospective screening survey was conducted from Feb. to June 2019 at the pediatric eye clinic of Menelik II Hospital. Children who were preterm at birth and who attended the eye clinic were included in the study. Data on demographic and neonatal characteristics, neonatal and maternal co-morbidities, and ocular disorders were collected. Odds ratio and univariate analysis were used to identify predictors of ocular diseases and visual impairment.

Results

There were 222 children included in the study with a mean age at presentation of 2.62 years (range 2.08- 6.38 years), mean GA 34.11 weeks (range 30-36) weeks, and mean birthweight 1941.72g (range 953-3500g). Nearly 2/3 had ocular disorders with refractive error (51.8%), strabismus (11.3%), and history of ROP (7.2%) being more common. One-fourth of the children had visual impairment, and the prevalence of amblyopia was 40.1%. Uncorrected refractive errors, strabismus, and ROP were causes for visual impairment.

Conclusion

Visual impairment and amblyopia are common in Ethiopia. There is a need to develop a screening protocol for ocular disorders for preterm children to enhance early detection and prevention of childhood visual impairment.

Keywords: Preterm children; Low birth weight; Visual Impairment; Refractive error; Strabismus; Retinopathy of prematurity; Risk factors of visual impairment; Sub-Saharan Africa; Ethiopia.

Confidential: For Review Only

Key Messages

What is already known about this subject?

- In many low- and middle-income countries, the survival of preterm infants has improved as neonatal systems have improved.
- Preterm children are at a higher risk of developing ocular disorders, visual impairment, and amblyopia than term children.

What this study adds

- The magnitude and causes of ocular morbidity among preterm children are not well studied in sub-Saharan African countries. This study, conducted among preterm children admitted to two NICUs in a sub-Saharan country, shows that preterm infants develop a higher rate of visual impairment and amblyopia.

How this study might affect research, practice or policy

- The findings of this study provide some evidence for screening for ocular diseases in preterm children, but further studies are needed.
- A follow up prospective study commencing in 5 years' time would be of value as the number of surviving very low birth weight infants may significantly increase

Confidential: For Review Only

INTRODUCTION

Global, regional, and national estimates of preterm birth (defined as childbirth at less than 37 completed weeks) using the 2019 Global Burden of Disease study showed 15.22 million preterm births (1). In the Global Burden of Disease Study, 3.1% of all disability-adjusted life-years were attributed to preterm birth, similar to the burden of HIV or malaria (2). More than 95% of preterm births are occurring in developing countries. Globally the estimated preterm birth rate is 11.1%. Over 60% of preterm births occur in Sub-Saharan Africa and South Asia (1). Ethiopia belongs to the top 15 countries that contribute to two-thirds of the world's preterm babies with a preterm rate of 14.1% out of 481 deliveries (3).

From six months of pregnancy to term is considered the most active period for ocular development (4). Improved neonatal care has increased the survival rates of extremely preterm infants with birth weights (BW) of 1,000 g or gestational age (GA) of 28 weeks; at the same time, retinopathy of prematurity (ROP) has become a significant threat to visual function (5-7). Preterm children are reported to have an increased incidence of visual impairment because of perinatal lesions in the brain (8-10).

It has been noted that both preterm birth and retinopathy of prematurity (ROP) have an effect on the developing visual system, leading to decreased visual acuity, decreased contrast sensitivity, and an increase in color vision deficiencies (11-16). Population-based studies suggest that ophthalmic impairments remain common in very low birth weight infants (11,16,17). Effects of prematurity on ocular and neurological development include retinopathy of prematurity (ROP), refractive error, strabismus, cerebral visual impairment, color vision deficits, reduced contrast sensitivity (CS), visual field defects, and decreased visual acuity (16). According to population studies, the incidence of ROP, whether moderate or severe, for infants born at less than 1500–1700 g ranges from 22–49% (17-19).

In a cohort study, children with lower birth weights had significantly worse near and distance visual acuity at ages 10 to 12 years compared to full-term infants (10). Additionally, infants born prematurely without ROP are more likely to have myopia and anisometropia than infants born at term because preterm babies are more likely to experience refractive errors (20). An increased incidence of strabismus has also been reported in children born prematurely, regardless of the presence of ROP (21-24).

Confidential: For Review Only

Research on ocular morbidities among preterm infants in sub-Saharan African nations is limited. Before 2020, blindness from ROP was not reported in Ethiopia, including studies in schools for the blind (25, 26).

To determine the top causes of illness and mortality in preterm infants admitted to neonatal intensive units (NICUs) in Ethiopia, an Ethiopian Study of Illness in Preterms (SIP) study was conducted based on standardized diagnostic protocols. This study is part of the SIP study focusing on ocular morbidities among preterms. The present study aimed to identify ocular disorders in a population of preterm children with and without ROP.

METHODS

Study design and subjects

The SIP Study is a prospective study conducted to determine the top causes of illness and mortality in preterm infants admitted to hospitals in Ethiopia based on standardized diagnostic protocols (27). The study participants of this current study are from the SIP study from Feb – June, 2019. The research was performed in accordance with the Declaration of Helsinki and was approved by the Institute Ethics Committee of Addis Ababa University ((Ref No. 003/2016). All parents or legal guardians provided informed consent before the examination. Patients or the public weren't involved in our research's design, conduct, reporting, or dissemination plans.

Study setting

For the SIP study, standard protocols were developed to undertake a physical examination and laboratory investigation, in particular microbiology, radiologic, and ultrasound examinations. There were initial and follow-up examinations to detect the progress of the preterm infant. Addis Ababa University, Gondar University, Jimma University, and St. Paul Millennium Medical College were included in the SIP study. However, for this ocular morbidity aspect of the SIP study, preterms from Addis Ababa, Tikur Anbessa Hospital, Gandhi Hospital, and St. Paul Millennium Medical College were included in the research.

Recruitment methods

Inclusion criteria were (1) GA < 37 weeks and (2) participation in the SIP study. The preterm children were identified from the SIP database. Parents of all preterm infants received a phone call invitation to participate in our investigation.

Confidential: For Review Only

Assessment of prenatal and postnatal history

History data were assessed from each child's recorded file for the enrolled children. The following data were extracted: antenatal risk factors: maternal age, in vitro fertilization, antenatal corticosteroids, preeclampsia/eclampsia, diabetes, HIV/AIDS, chorioamnionitis, mode of delivery, and multiple births. Neonatal factors included sex, GA, BW, resuscitation in the delivery room, respiratory distress syndrome (RDS), duration of invasive/noninvasive mechanical ventilation and oxygen therapy, intracranial hemorrhage, patent ductus arteriosus (PDA), neonatal sepsis, necrotizing enterocolitis (NEC), number of blood transfusions, and bronchopulmonary dysplasia (BPD). There were no regular ROP screening programmes within the NICUs of the hospitals where the patients were admitted. There was no referral system from the NICUs to Ophthalmology clinic, except if the parents noted a concern. In addition, all parents were interviewed using a standardized protocol to request information concerning medical history of the child and parents, including ocular and general morbidities.

Definitions

Gestational age was determined using last menstrual period [LPM], Ballard and Dubowitz scores and ultrasound assessment. Studies in Papua New Guinea have shown good concordance (0.878, 0.914, and 0.886, respectively) compared to antenatal ultrasound as the gold standard (28). LMP in a low-resource setting such as Bangladesh was found to be a more reliable measure of gestational age than previously thought for the estimation of postnatal gestational age of preterm infants (29).

Preterm infants were further classified as late and moderate preterm (32 to < 37 weeks), very preterm (28 to <32 weeks), and extremely preterm (less than 28 weeks). Glasses were prescribed if there was myopia >1.0D, astigmatism >1.0D, or hypermetropia >+2.0D.

Eye examination

All examinations were performed by the PI and lead author (STS), a pediatric ophthalmologist. Testing of best-corrected visual acuity was performed with Lea symbols until school enrolment, and after that, ETDRS was used in all subjects. In cases of visual acuity below 6/60, depending on the children's age, Lea symbols or Landolt rings were used at a distance of 1 m. Values were converted for analysis into the logarithm of the minimum angle of resolution (logMAR) (30).

https://mc.manuscriptcentral.com/bmjpo

Cyclopentolate (0.5%) eye drops were administered three times at 10-min intervals, after which cycloplegic refraction and keratometry were analyzed with an autorefractor (Nidek ARK-1s keratometer, Japan). The spherical equivalent (refractive error) was calculated by adding the spherical value and half of the cylindrical value. Anisometropia was defined as a difference between the patients' eyes of ≥ 1.5 diopters of spherical equivalent. Orthoptic examination for strabismus included the cover-uncover test and alternate cover test, the Hirschberg Test and examination of fixation behavior, as well as the presence or absence of nystagmus after having corrected refractive errors. If a child presented with heterotropia, an alternating prism cover test was added to measure the angle of deviation in prism diopters.

Strabismus was defined as constant or intermittent heterotropia of any dimension at a distance and/or near fixation after correcting refractive error. Classification of strabismus was categorized depending on deviation from the primary position (esotropia or exotropia). An anterior segment examination was done using slit lamp biomicroscopy. A dilated posterior segment examination was conducted using indirect ophthalmoscopy with a 28-diopter lens. Retinopathy of prematurity was diagnosed retrospectively from the patients' chart.

Data analyzed using IBM SPSS 21.0 (SPSS Inc., Chicago, USA). Continuous variables were expressed as the mean ± standard deviation (SD) or as the median when appropriate. Categorical variables were expressed as proportions. The chi-square test was used to analyze the association between categorical variables. Associations between ocular morbidities and continuous and categorical variables were computed using Fisher's exact test and Pearson chi-square (χ^2) test, respectively. Continuous variables were compared using ANOVA. Values of $p < 0.05$ were considered statistically significant.

For Review Only

RESULTS

During the study period 222 infants (146 from Saint Paul Hospital and 76 from TASH) were included in this study.

Characteristics of the study population

Slightly more females than males were screened (52.7% and 47.3%, respectively). The majority of the study participants (n=156, 70.3%) were less than 3 years of age and the mean age at presentation was 2.62 ± 0.49 years (range 2.08- 6.38). One hundred and twenty-three of the 222 children (55.4%) had a GA ≤34 weeks and 43 (19.4%) had a BW ≤1500g. Birthweight ranged from 953-3500g with a mean of 1941.72g (SD 445.49); GA ranged from 30-36 weeks, with a mean of 34.11 weeks (SD 1.47). One hundred and twenty-three children (55.4%) were delivered vaginally, and 80 (36.1%) had multiple gestations. Forty-eight children (21.7%) were born to mothers with pregnancy-induced hypertension, and eight (3.7%) mothers tested positive for HIV (Table 1).

The mean BWs of children from SPH and TASH NICUs were 1888.5 ± 403.6 (953-3000) g and 2043.94 ± 503.74 (1125-3500) g, respectively; mean GAs were 34.14 ± 1.49 (30-36) weeks and 34.08 ± 1.44 (30-36) weeks, respectively. Differences in these parameters were not statistically significant (Table 2).

Confidential: For Review Only

Table 1 Characteristics of premature children and mothers screened for ocular disorders

Variable		Total	Male		Female	
		N	N	%	N	%
**Birthweight**	≤1500 gm	43	20	46.5%	23	53.5%
	>1500gm	179	85	47.5%	94	52.5%
**Gestational age**	≤34 weeks	122	60	49.2%	62	50.8%
	> 34 weeks	100	45	45%	55	55%
**Multiple gestation**	Yes	80	42	52.5%	38	47.5%
	No	142	63	44.4%	79	55.6%
**Oxygen supplementation**	Yes	97	47	48.5%	50	51.5%
	No	125	58	46.4%	67	53.6%
**Infantile morbidity**	Sepsis	6	2	33.3%	4	66.7%
	IVH	2	0	0%	2	100%
	BPD and Sepsis	1	1	100%	0	0%
	None	213	102	47.9%	111	52.1%
**Mode of delivery**	Vaginal delivery	123	53	43.1%	70	56.9%
	Cesarean section	99	52	52.5%	47	47.5%
**Multiparity**	Yes	47	18	38.3%	29	61.7%
	No	175	63	34.3	59	65.7%
**Maternal morbidity**	PIH	44	22	50%	22	50%
	HIV	5	2	40%	3	60%
	HIV & PIH	3	3	100%	0	0%
	DM	2	0	0%	2	100%
	DM & PIH	1	0	0%	1	100%
	TORCH	1	1	100%	0	0%
	None	166	77	46.4%	89	53.6%
**NICU location**	SPH	146	65	44.5%	81	55.5%
	TASH	76	40	52,6%	36	47.4%

Legend: BPD- Bronchopulmonary Dysplasia; DM- Diabetes Mellitus; HIV-IVH-Intraventricular Hemorrhage; PIH-Pregnancy-Induced Hypertension; SPH- Saint Paul Hospital; TASH-Tikur Anbessa Specialized Hospital; TORCH-Toxoplasmosis, Rubella, Cytomegalovirus, Herpes simplex, and HIV

Ocular morbidities and risk factors

Overall, 145 (65.3%) of the children had ocular disorders at the presentation, of which 92 (63.4%) had isolated ocular diseases (69 refractive error, 13 nasolacrimal duct obstruction, five strabismus, and five ROP). The mean age at presentation of children with ocular disorders was 2.7±0.5 (2.1- 6.4) years, and there were more females with a male to female ratio of 1:1.27. None of the eyes examined had anomalies of the anterior segment or lens.

The mean GA was 34.14 ± 1.49 (30-36) weeks, and BW was 1927.27 ± 429.19 (953-3100) grams. Refractive errors were the leading type of ocular morbidity seen in 115/222 (51.8%), followed by NLDO (21.2%) (Table 2).

Confidential: For Review Only

Table 2. Types of ocular disorders among premature children screened

Ocular disorders	n	%
Refractive error	115	51.8
Nasolacrimal duct obstruction	47	21.2
Strabismus	25	11.3
Retinopathy of prematurity	16	7.2
Others	5	2.3

NB- Some ocular disorders occur more than once.

Refractive error

One hundred and fifteen (51.8) children had a refractive error, of which 55.5% (81/146) and 44.7% (34/76) of children enrolled from the SPH and TASH had refractive errors, respectively. The mean age at presentation was 2.68 ±0.56 (2.08-6.38) years, and the male-to-female ratio was 1:1.25. Thirty-nine (59%) of children aged > 3 years developed refractive error in comparison with 76 (48.1%) of those aged < 3 years.

The mean gestational age and birthweight of children with refractive errors was 34.11±1.54 (30-36) weeks and 1892.34±414.55 (1080-3100) grams, respectively. Myopia was the commonest type of refractive error, accounting for 78/115 (60.8%) of cases, followed by astigmatism (30, 26.1%) and hyperopia (15, 13.1%). Gender, GA, BW, oxygen supplementation, children, and maternal morbidity were not statistically associated with refractive error. (Table 3)

Strabismus

Twenty-five children (11.3%) had strabismus (5 isolated, 20 in combination with refractive error, nystagmus, ROP, and nasolacrimal duct obstruction). The age at presentation was 2.73±0.52 (2.1-3.6) years, and the male-to-female ratio was 1.08:1.

The mean GA and BW were 34.0±1.41 (30-36) weeks and 906.76±489.92 (1140-3000), respectively. Regarding the types of strabismus, 13 cases had esotropia, and the remaining 12 patients had exotropia. There was no statistically significant association between GA, BW, and strabismus (Table 3). In this study, the prevalence of strabismus among children aged ≥ 3years was 16.7% compared to 8.9% in those < 3 years. However, older age was not statistically associated with strabismus.

Confidential: For Review Only

Retinopathy of prematurity

Previous history of ROP was noted in 16/22 (7.2%) of the children enrolled in this study. Most patients (12, 57%) with ROP were from SPH. Almost all of them (15/16) had a GA < 34 weeks, and the mean GA and BW of patients with ROP were 32.19±1.33 (30-35) weeks and 1596.25 ±483.64 (953-2600) grams, respectively. Nine patients with ROP had an associated refractive error (6 myopia and 3 astigmatism). Only one patient had an associated intermittent exotropia. In univariate analysis, ROP was statistically associated with low GA and low BW (Table 3). Multivariable logistic regression analysis was not conducted due to the small number of children with ROP.

Table 3. Ocular disorders by sex, gestational age, and birthweight among premature children screened for ocular disorders

Type of Disorder	Variables		Yes	No	Odds Ratio	p-Value
			n	n		
Refractive error	Sex	Male	51	54	0.78(0.46-1.33)	0.361
		Female	64	53		
	BWt	≤1500 gm	22	21	1.03(0.53-2.01)	0.926
		>1500gm	93	86		
	GA	≤34 weeks	62	60	1.09)0.64-1.85)	0.746
		> 34 weeks	53	47		
Strabismus	Sex	Male	12	93	1.03(0.4501.37)	0.940
		Female	13	104		
	BWt	≤1500 gm	7	36	0.57(0.22-1.48)	0.246
		>1500gm	18	161		
	GA	≤34 weeks	14	108	0.95 (0.41-2.2)	0.911
		> 34 weeks	11	89		
ROP	Sex	Male	6	99	0.65(0.22-1.85)	0.415
		Female	10	107		
	BWt	≤1500 gm	7	36	Reference	0.01
		>1500gm	9	170	0.27(0.09-0.78)	
	GA	≤34 weeks	15	107	Reference	0.001
		> 34 weeks	1	99	0.72 (0.09-0.56)	

Legend: BWt- Birth Weight ; GA-Gestational Age

Visual impairment and ocular disorders

The mean VA of the right and left eyes was 0.22(SD 0.23) logMAR and 0.17 (SD 0.21) logMAR, respectively. The mean VA in the better and worse eyes was 0.17 (SD 0.22) logMAR and 0.28 (SD 0.21) logMAR, respectively. In this study, 101 (45.9%) and 181 (81.5%) of the children had subnormal visual acuity (>logMAR 0.1) in the better and worst eyes, respectively.

Confidential: For Review Only

Nearly one-fourth (55, 24.8%) of children screened had visual impairment in the better eye. Of this group 51 (92.7%) had uncorrected refractive error alone (34/51) or with strabismus (10/51), ROP (6/51), or nystagmus (1/51). Eighty-nine (40.1%) patients had amblyopia, of which 59/89 (66.3%) had bilateral amblyopia from uncorrected refractive error. Isometropic and anisometropic amblyopia from uncorrected refractive error were the commonest causes of amblyopia, contributing to 49/89 (55%) and 20/89 (22.8%), respectively. Of the 16 cases with ROP, 12 (75%) had a visual impairment associated with other disorders like refractive error, strabismus, and nystagmus.

In univariate analysis, visual impairment in the better eye was statistically associated with ROP, uncorrected refractive error, and strabismus with p-values of 0.001, 0.001, and 0.004, respectively. Amblyopia was not statistically associated with low GA or low BW (Table 4).

Table 4. The presence of visual impairment by types of ocular disorders among premature children screened for ocular disorders.

Variables		Visual Impairment in the better eye		Odds Ratio 95% CI (Lower-Upper)	p-Value
		Yes	No		
Sex	Male	23	82	0.75(0.40-1.38)	0.348
	Female	32	85		
**BWt**	≤1500 gm	14	29	0.62(0.29-1.27)	0.188
	>1500gm	41	138		
**GA**	≤34 weeks	28	94	1.24(0.67-2.29)	0.294
	> 34 weeks	27	73		
**Refractive error**	Yes	48	67	10.24(4.37-23.97)	0.001
	No	7	100		
**Strabismus**	Yes	12	13	3.31 (1.41-7.77)	0.004
	No	43	154		

Confidential: For Review Only

DISCUSSION

The present prospective study examines the effects of prematurity on visual acuity and ocular disorder in children born preterm. In Sub-Saharan Africa, neonatal death has decreased by 40% since 1990 due to improved newborn care, likely leading to an increase in childhood ocular morbidity and blindness from diseases like ROP (32). Despite this positive progress, data on the extent of ocular diseases among the preterms in Sub-Saharan Africa are limited. Our study has demonstrated that the prevalence of ocular diseases and visual impairment in Ethiopian children born preterm is high. To our knowledge, this is the first study to assess the prevalence and causes of ocular disorders and visual impairment among children born preterm and admitted to NICUs. In Ethiopia, intensive neonatal care has expanded in many public and private hospital NICUs since 2013 (33), and neonatal mortality per thousand live births has declined modestly from 39 in 2000 to 33 in 2019(34). A prospective screening survey among neonates admitted to two NICUs in Ethiopia showed that 32.2% of the screened infants had any stage ROP (35). However, there is no regular ROP screening program in the country. A comparison of studies of ocular morbidity and visual impairment among preterm children is difficult as there are methodological variations such as differing age groups, inclusion or exclusion of ROP, stages of ROP, and cohort size. Even though genetic and visual experiences predominantly determine the prevalence of refractive error, studies have shown that low BW interrupts emmetropisation and increases the prevalence of refractive error (36). In our study, nearly half of the premature screened children (51.8%) had refractive error, which is comparable to a survey from Turkey (53.8%) (37) but higher than in Italy (42.3%) (38), and in cohorts of extremely preterm infants from Sweden (29.7%) (39) and Norway (10%) (40). In our study, the prevalence of myopia was 35.1%, which was higher than a cohort of preterm children at age 10–12 years from the UK (18.9%) (41), India (15.8%) (42), and Sweden (4.1%) (39). The prevalence of hyperopia in our study, 13.5%, is comparable with that reported in Turkey (14.3%) (37) and Sweden (17.1%) (39) but higher than the UK (6.6%) (41) and India (8.54%) (42). In the present study, 13.5% of the preterm children had clinically significant astigmatism, which was lower than that reported in Norway (21%) (40) and India (55.6%) (42) and higher than in Turkey (5.7%) (37) and Sweden (6.5%) (39). The higher proportion of myopia seen in our study, in comparison with studies from the UK (41),

Confidential: For Review Only

India (42) and Sweden (39), is supported by long-term studies which have confirmed the increased incidence of myopia following preterm birth (43).

Manifest strabismus was seen in 11.3% of our cohort, which is comparable to studies from Norway (10%) (40), the UK (13.6%) (15), Sweden (13.5%) (20), and Australia (14%) (45), and lower than reported in Sweden (17%) (36), the UK and Ireland (24%) *(46)* and Germany (26%) (47). It is unclear at what age the different types of strabismus develop (36), and the age at onset of strabismus in low birthweight children is variable, from the first few months of life to many years later (11, 15, 16,21,22, 44). In our study, a higher prevalence of strabismus in those aged > 3 years was noted. This finding (16.7%) is comparable with a similar age group from Sweden (20). Regarding the type of strabismus, we detected similar proportions for esotropia and exotropia. This is similar to the other studies from Germany (47) and England (41). However, other investigations confirmed that esotropia was the most frequent type of strabismus (20, 39, 48) The increased prevalence of strabismus in the low birthweight population is well-documented (21, 36, and 44). Such an association was not apparent in our study, as most of the children were considerably higher in weight and older than in the studies mentioned above.

The prevalence of ROP in our study is 7.2%, lower than in other studies, from sub-Saharan African countries, including Ethiopia, which ranged from 15-41.7% (35, 49-51). The lower prevalence of ROP in our study can be explained by our data collection method, where we depend on the history of ROP either from the patient's parents or from old features of ROP.

In the present study, 46% of the children had subnormal visual acuity (>logMAR 0.1) in the better eye, which is comparable with a population-based study from Norway (45.9%) (40). The figure is higher than what has been reported for prematurely-born children with BWs 1500–2000 g (15 %) from Denmark (9) and from Sweden 32 % (20). Birch et al. reported significantly lower visual acuities in low birthweight infants compared to those born full term (52). In our study, there was no statistically significant correlation between BCVA and BW or GA, similar to a study from Turkey (37). However, Dowdeswell et al. (53) found low levels of distance visual acuity in preterm children compared with full-term children. However, in our study, ocular morbidities like strabismus, refractive error, and ROP were statistically associated with visual impairment.

In our study, the prevalence of amblyopia among premature children was 40.1%. The result in our study is much higher than other studies from Australia (7.3%) (45) and Turkey

Confidential: For Review Only

(7.7%) (37). Previous studies have shown that prematurity and low birthweight are two risk factors for amblyopia (41, 54). Nevertheless, amblyopia was not statistically associated with low GA and BW. Even if we did not find a statistical association between GA and BW with amblyopia, the prevalence among premature children is higher than in other studies; this indicates that more importance should be given to screening amblyopia risk factors for premature infants.

The strengths of this study were the prospective controlled study design with a high number of participants, the multi-center design which increases the representativeness of our research, and the availability of medical information from all children and mothers, which allowed a very detailed examination and an adjustment for different possible confounding factors. The strict standardization reduced the probability of examiner-dependent variances.

Limitations of the study included the wide age range of the examined children, some of whom were at an early age and phase of refractive development, and other older children that can affect the physiologic refractive changes noted in normal health children. The other limitation is there is a chance that those infants with poor health outcomes did not take part in our study. In subsequent research, we will continue following up with these infants to determine future changes in their refractive error and strabismus.

CONCLUSION

In conclusion, the rates of ocular disorders, visual impairment, and amblyopia in these NICUs in Ethiopia were higher than in other studies. Refractive error, strabismus, and ROP were all significant risk factors for visual impairment. These findings underline the importance of early screening of premature infants for vision and amblyopia. As the two NICUs included in the survey are Ethiopia's main neonatal referral centers, it can be postulated that ocular morbidities, visual impairment, and amblyopia are emerging as potentially avoidable causes of childhood blindness among preterm children in Ethiopia. Developing preterm ocular-related screening protocols within the NICUs, strengthening the referral links between the NICUs and eye centers, and further detailed comparative studies between preterm and term children for ocular disorders are recommended.

Confidential: For Review Only

Lists of Abbreviations

Bronchopulmonary dysplasia (BPD); Necrotizing enterocolitis (NEC); Neonatal Intensive Care Units (NICUs); Patent ductus arteriosus (PDA); Preterm birth (PTBs); Respiratory distress syndrome (RDS); Retinopathy of prematurity (ROP), Saint Paul Hospital (SPH) and Tikur Anbessa Specialized Hospitals (TASH).

Research Ethics Approval

This study involves human participants, and this research was approved by the ethics committee of Addis Ababa University Ethics Review Committee (Ref No. 003/2016) in line with the relevant national and institutional guidelines on care and clinical research. All parents or legal guardians gave written informed consent before participating in the study.

Availability of Data and Materials

All data generated or analysed during this study are included in this published article

Competing Interests

The authors have no conflicts of interest.

Funding

The SIP study was supported by the Bill & Melinda Gates Foundation (OPP1136965)

Authors' Contributions

Drafting of the manuscript: STS., LM., AM. , and AD. Revision of the manuscript for important intellectual content: STS., LM., AM., AD and AA. Conception and design of study: STS., LM., AM. , and AD. Data acquisition, analysis, or interpretation of data: STS., LM., AM., AD., and AA. Approval of final manuscript to be published: STS., LM., AM., AD., and AA. All authors have read and approved the final version of the manuscript.

Acknowledgements

The authors wish to acknowledge the assistance of the staff of the SIP project (Ahmed, Beleyu, Efrata, and Wagaye) for their support during data collection. Our special appreciation goes to Sr Martha H/Mariam and Sr. Medhanit from Menelik II pediatrics Ophthalmology clinic.

Confidential: For Review Only

REFERENCES

1. Cao G, Liu J, Liu M. Global, regional, and national incidence and mortality of neonatal preterm birth, 1990-2019. JAMA pediatrics. 2022 Aug 1;176(8):787-96.

2. World Health Organization. 2014. Global Burden of Diseases Estimates http://www.who.int/healthinfo/global_burden_disease/en/.

3. Adane AA, Ayele TA, Ararsa LG, Bitew BD, Zeleke BM. Adverse birth outcomes among deliveries at Gondar University Hospital, Northwest Ethiopia, BMC Pregnancy and Childbirth 2014, 14:90.

4. Hellström A, Smith LE, Dammann O. Retinopathy of prematurity. The lancet. 2013 Oct 26;382(9902):1445-57.

5. Gogate P, Gilbert C, Zin A. Severe visual impairment and blindness in infants: causes and opportunities for control. Middle East Afr J Ophthalmol. 2011;18(2):109–114.

6. Chen C, Zhang QS. [Advances in medical care for extremely low birth weight infants worldwide]. Zhongguo Dang Dai Er Ke Za Zhi. 2013;15(8):703–707. Chinese.

7. Mccolm JR, Fleck BW. Retinopathy of prematurity: causation. Semin Neonatol. 2001;6(6):453–460.

8. Cats BP, Tan KEWP. Prematures with and without regressed retinopathy of prematurity: comparison of long-term (6–10 years) ophthalmological morbidity. J Pediatr Ophthalmol Strab 1989;Nov/Dec:271–5.

9. Fledelius HC, Greisen G. Very pre-term birth and visual impairment. Acta Ophthalmol 1993;Suppl 210:63–5.

10. Gallo JE, Lennerstrand G. A population-based study of ocular abnormalities in premature children aged 5 to 10 years. Am J Ophthalmol 1991;111:539–47.

11. Holmstrom G, el Azzazi M, Kugelberg U. Ophthalmological follow up of preterm infants: a population based, prospective study of visual acuity and strabismus. Br J Ophthalmol 1999;83:143–50.

12. Sebris SL, Dobson V, Hartmann EE. Assessment and prediction of visual acuity in 3-to 4-year-old children born prior to term. Human Neurobiol 1984;3:87–92.

13. Visual function in low birthweight children A R O'Connor, T J Stephenson, A Johnson, M J Tobin, S Ratib, M Moseley, A R Fielder.

Confidential: For Review Only

14. Larsson E, Rydberg A, Holmstrom G. Contrast sensitivity in 10 year old preterm and full term children: a population basedstudy. Br J Ophthalmol 2006;90:87–90.
15. Cooke RW, Foulder-Hughes L, Newsham D, Clarke D. Ophthalmic impairment at 7 years of age in children born very preterm. Arch Dis Child Fetal Neonatal Ed 2004;89:F249–53.
16. O'Connor AR, Stephenson TJ, Johnson A, Tobin MJ, Ratib S, Moseley M, et al. Visual function in low birthweight children. Br J Ophthalmol 2004;88:1149–53.
17. Darlow BA, Clemett RS, Horwood LJ, et al. Prospective study of New Zealand infants with birth weight less than 1500 g and screened for retinopathy of prematurity: visual outcome at age 7–8 years. Br J Ophthalmol 1997;81:935–40.
18. Ng YK, Fielder AR, Shaw DE, Levene MI. Epidemiology of retinopathy of prematurity. Lancet 1988; 2(8622): 1235–1238.
19. Holmstrom G, el Azazi M, Jacobson L, Sachs D, Sule J, Lennerstrand G. Epidemiology of ROP in the Stockholm area of Sweden. Acta Opthalmol 1993; 210(Suppl): 44–47.
20. Holmström G, El Azazi M, Kugelberg U. Ophthalmological long term follow up of preterm infants: a population based, prospective study of the refraction and its development. Br J Ophthalmol. 1998;82:1265–1271.
21. Laws D, Shaw DE, Robinson J, et al. Retinopathy of prematurity: a prospective study. Review at six months. Eye 1992;6:477–83.
22. Pennefather PM, Clarke MP, Strong NP, et al. Ocular outcome in children born before 32 weeks gestation. Eye 1995;9(Suppl):26–30.
23. Robinson R, O’Keefe M. Follow-up study on premature infants with and without retinopathy of prematurity. Br J Ophthalmol 1993;77:91–4.
24. McGinnity FG, Bryars JH. Controlled study of ocular morbidity in school children born preterm. Br J Ophthalmol 1992;76:520–4.
25. Asferaw M, Woodruff G, Gilbert C. Causes of severe visual impairment and blindness in students in schools for the blind in Northwest Ethiopia. BMJ Glob Health. 2017;2(2):e000264.
26. Kello AB, Gilbert C. Causes of severe visual impairment and blindness in children in schools for the blind in Ethiopia. Br J Ophthalmol. 2003;87(5):526-30.
27. Muhe, L.M., McClure, E.M., Mekasha, A. et al. A Prospective Study of Causes of Illness and Death in Preterm Infants in Ethiopia: The SIP Study Protocol. Reprod Health 15, 116 (2018). https://doi.org/10.1186/s12978-018-0555-y.

Confidential: For Review Only

28. Karl S. Connie SN, Wai Suen et al. 2015. Preterm or Not – An Evaluation of Estimates of Gestational Age in a Cohort ofWomen from Rural Papua New Guinea. PLOSONE |DOI:10.1371/journal.pone.0124286 May6, 2015.

29. Rosenberg RE, Nawshad ASM, Ahmed U. et al 2009. Determining Gestational Age in a Low-resource Setting: Validity of Last Menstrual Period. J HEALTH POPUL NUTR 2009 Jun;27(3):332-338.

30. Bach M, Kommerell G. Determining visual acuity using European normal values: scientific principles and possibilities for automatic measurement. Klin Monatsbl Augenheilkd. 1998;212(4):190–5.

31. Hosmer DW, Lemesbow S. Goodness of fit tests for the multiple logistic regression model. Communications in statistics-Theory and Methods. 1980 Jan 1;9(10):1043-69.

32. Hug L, Alexander M, You D, Alkema L. National, regional, and global levels and trends in neonatal mortality between 1990 and 2017, with scenario-based projections to 2030: a systematic analysis. The Lancet Global Health. 2019 Jun 1;7(6):e710-20.

33. UNICEF. Investing in Survival: Enhancing the Neonatal Intensive Care Unit of Yekatit 12 Hospital: A Final Report for UNICEF's Next Generation; 2013 file. https://www.unicefusa.org/sites/default/files/Ethiopia%20Report.pdf. Accessed January 2023.

34. Ministry of Health-Ethiopia. Health Sector Transformation Plan-II. 2021. https://www.globalfinancingfacility.org/ethiopia-health-sector-transformation-plan-201920-202425. Accessed January 2023.

35. Sherief ST, Taye K, Teshome T, Demtse A, Gilbert C. Retinopathy of prematurity among infants admitted to two neonatal intensive care units in Ethiopia. BMJ Open Ophthalmology. 2023 Jul 1;8(1):e001257.

36. O'Connor A, Fielder AR. Long term ophthalmic sequelae of prematurity. Early human development. 2008 Feb 1;84(2):101-6.

37. Özdemir M, Koylu S. Ocular growth and morbidity in preterm children without retinopathy of prematurity. Japanese journal of ophthalmology. 2009 Nov;53:623-8.

38. Ricci B. Refractive errors and ocular motility disorders in preterm babies with and without retinopathy of prematurity. Ophthalmologica. 1999 Oct 1;213(5):295-9.

39. Hellgren KM, Tornqvist K, Jakobsson PG, Lundgren P, Carlsson B, Källén K, Serenius F, Hellström A, Holmström G. Ophthalmologic outcome of extremely preterm infants at 6.5 years

Confidential: For Review Only

of age: Extremely Preterm Infants in Sweden Study (EXPRESS). JAMA ophthalmology. 2016 May 1;134(5):555-62.

40. Haugen OH, Nepstad L, Standal OA, Elgen I, Markestad T. Visual function in 6 to 7 year-old children born extremely preterm: a population-based study. Acta ophthalmologica. 2012 Aug;90(5):422-7.

41. O'Connor AR, Stephenson TJ, Johnson A, Tobin MJ, Ratib S, Fielder AR. Change of refractive state and eye size in children of birth weight less than 1701 g. British journal of ophthalmology. 2006 Apr 1;90(4):456-60.

42. Sathar A, Abbas S, Nujum ZT, Benson JL, Sreedevi GP, Saraswathyamma SK. Visual outcome of preterm infants screened in a tertiary care hospital. Middle East African Journal of Ophthalmology. 2019 Jul;26(3):158.

43. O'Connor AR, Wilson CM, Fielder AR. Ophthalmological problems associated with preterm birth. Eye. 2007 Oct;21(10):1254-60.

44. Theng JT, Wong TY, Ling Y. Refractive errors and strabismus in premature Asian infants with and without retinopathy of prematurity. Singap Med J 2000;41:393–7.

45. Hebbandi SB, Bowen JR, Hipwell GC, Ma PJ, Leslie GI, Arnold JD. Ocular sequelae in extremely premature infants at 5 years of age. Journal of paediatrics and child health. 1997 Aug;33(4):339-42.

46. Marlow N,Wolke D, BracewellMA, SamaraM; EPICure Study Group. Neurologic and developmental disability at six years of age after extremely preterm birth. N Engl J Med. 2005;352(1):9-19.

47. Fieß A, Kölb-Keerl R, Schuster AK, Knuf M, Kirchhof B, Muether PS, Bauer J. Prevalence and associated factors of strabismus in former preterm and full-term infants between 4 and 10 Years of age. BMC ophthalmology. 2017 Dec;17(1):1-9.

48.VanderVeen DK, Bremer DL, Fellows RR, Hardy RJ, Neely DE, Palmer EA, Rogers DL, Tung B, Good WV, Early Treatment for Retinopathy of Prematurity Cooperative G. Prevalence and course of strabismus through age 6 years in participants of the early treatment for retinopathy of prematurity randomized trial. J AAPOS. 2011;15(6):536–40.

49. Fajolu IB, Rotimi-Samuel A, Aribaba OT, et al. Retinopathy of prematurity and associated factors in Lagos, Nigeria. Paediatr Int Child Health. 2015;35(4):324-8.

Confidential: For Review Only

50. Omer IM, Hassan HA. The prevalence and risk factors of retinopathy of prematurity among preterm babies admitted to Soba Neonatal Intensive Care Unit. Sudan J Paed. 2014;14(2):17.

51. Wanjala I ID, Kariuki L. Retinopathy of Prematurity as seen in two major hospitals in Nairobi, Kenya. East Afr J Ophthalmol. 2007 May;13(1):5-14.

52. Birch EE, O'Connor AR. Preterm birth and visual development. InSeminars in Neonatology 2001 Dec 1 (Vol. 6, No. 6, pp. 487-497). WB Saunders.

53. Dowdeswell HJ, Slater AM, Broomhall J, Tripp J. Visual defi cits in children born at less than 32 weeks gestation with and without major ocular pathology and cerebral damage. Br J Ophthalmol 1995;79:447–452.

54. Mocanu V, Horhat R. Prevalence and risk factors of amblyopia among refractive errors in an Eastern European population. Medicina. 2018 Mar 20;54(1):6.

Confidential: For Review Only

TABLES LEGEND

Table 1 Characteristics of premature children and mothers screened for ocular disorders

Table 2. Types of ocular disorders among premature children screened

Table 3. Ocular disorders by sex, gestational age, and birthweight among premature children screened for ocular disorders

Table 4. The presence of visual impairment by types of ocular disorders among premature children screened for ocular disorders.

Confidential: For Review Only

BMJ Paediatrics Open

Prevalence and causes of ocular disorders and visual impairment among preterm children in Ethiopia

Journal:	*BMJ Paediatrics Open*
Manuscript ID	bmjpo-2023-002317.R3
Article Type:	Original research
Date Submitted by the Author:	15-Jan-2024

Complete List of Authors:	SHERIEF, SADIK; Addis Ababa University, Department of Ophthalmology; SickKids Research Institute<br>Muhe, Lulu M; Addis Ababa University College of Health Sciences<br>Mekasha, Amha; Addis Ababa University, Department of Pediatrics and Child Health<br>Demtse, Asrat; Addis Ababa University College of Health Sciences, Paediatrics and Child Health<br>Ali, Asim; The Hospital for Sick Children, Ophthalmology and Vision Sciences; University of Toronto, Department of Ophthalmology and Vision Sciences

Keywords:	Infant, Neonatology, Ophthalmology

SCHOLARONE™
Manuscripts

I, the Submitting Author has the right to grant and does grant on behalf of all authors of the Work (as defined in the below author licence), an exclusive licence and/or a non-exclusive licence for contributions from authors who are: i) UK Crown employees; ii) where BMJ has agreed a CC-BY licence shall apply, and/or iii) in accordance with the terms applicable for US Federal Government officers or employees acting as part of their official duties; on a worldwide, perpetual, irrevocable, royalty-free basis to BMJ Publishing Group Ltd ("BMJ") its licensees and where the relevant Journal is co-owned by BMJ to the co-owners of the Journal, to publish the Work in this journal and any other BMJ products and to exploit all rights, as set out in our licence.

The Submitting Author accepts and understands that any supply made under these terms is made by BMJ to the Submitting Author unless you are acting as an employee on behalf of your employer or a postgraduate student of an affiliated institution which is paying any applicable article publishing charge ("APC") for Open Access articles. Where the Submitting Author wishes to make the Work available on an Open Access basis (and intends to pay the relevant APC), the terms of reuse of such Open Access shall be governed by a Creative Commons licence – details of these licences and which Creative Commons licence will apply to this Work are set out in our licence referred to above.

Other than as permitted in any relevant BMJ Author's Self Archiving Policies, I confirm this Work has not been accepted for publication elsewhere, is not being considered for publication elsewhere and does not duplicate material already published. I confirm all authors consent to publication of this Work and authorise the granting of this licence.

Confidential: For Review Only

Prevalence and causes of ocular disorders and visual impairment among preterm children in Ethiopia

Sadik Taju Sherief [1,2], Lulu Muhe [3] Amha Mekasha [3], Asrat Demtse [3], and Asim Ali[4,5]

[1] Department of Ophthalmology, Addis Ababa University, Addis Ababa, Ethiopia;

[2] Child Health Evaluative Sciences Program and Centre for Global Child Health, Sickkids Research Institute, Toronto, Canada;

[3] Department of Pediatrics and Child Health, Addis Ababa University, Addis Ababa, Ethiopia;

[4] Department of Ophthalmology and Vision Sciences, The Hospital for Sick Children, Toronto, Canada.

[5] Department of Ophthalmology and Vision Sciences, University of Toronto, Toronto, Canada.

Short Title: Ocular disorders in Preterms

Corresponding Author:
Sadik T. Sherief, MD
Department of Ophthalmology
School of Medicine, College of Health Sciences
Addis Ababa University
P.O. Box 9086, Addis Ababa, Ethiopia
Tel: +251917801719
Email: goge4000@yahoo.com

Submitted as an Original Research article to: BMJ Paediatrics Open

Abstract word count (300 maximum): 235 words

Manuscript word count (4000 maximum): 3,433 words

Tables and Figures (maximum 5): 4 Tables

Reference Count: 54

Submitted to: *BMJ Paediatrics Open*

Key words: Preterm children; Low birth weight; Visual Impairment; Refractive error; Strabismus; Retinopathy of prematurity; Risk factors of visual impairment; Sub-Saharan Africa; Ethiopia.

Confidential: For Review Only

ABSTRACT

Objective

The aim of this study was to determine the prevalence, causes of ocular disorders and visual impairment among preterm children previously admitted to neonatal intensive care units in Addis Ababa, Ethiopia.

Methods and Analysis

A prospective screening survey was conducted from February to June 2019 at the pediatric eye clinic of Menelik II Hospital. Children who were preterm at birth and who attended the eye clinic were included in the study. Data on demographic and neonatal characteristics, neonatal and maternal co-morbidities, and ocular disorders were collected. Odds ratio and univariate analysis were used to identify predictors of ocular diseases and visual impairment.

Results

There were 222 children included in the study with a mean age at presentation of 2.62 years (range 2.08- 6.38 years), mean gestational age (GA) 34.11 weeks (range 30-36) weeks, and mean birthweight 1941.72g (range 953-3500g). Nearly 2/3 had ocular disorders with refractive error (51.8%), strabismus (11.3%), and history of retinopathy of prematurity (ROP) (7.2%) being more common. One-fourth of the children had visual impairment, and the prevalence of amblyopia was 40.1%. Uncorrected refractive errors, strabismus, and ROP were causes for visual impairment.

Conclusion

Visual impairment and amblyopia are common in Ethiopia. There is a need to develop a screening protocol for ocular disorders for preterm children to enhance early detection and prevention of childhood visual impairment.

Keywords: Preterm children; Low birth weight; Visual Impairment; Refractive error; Strabismus; Retinopathy of prematurity; Risk factors of visual impairment; Sub-Saharan Africa; Ethiopia.

Confidential: For Review Only

Key Messages

What is already known about this subject?

- In many low- and middle-income countries, the survival of preterm infants has improved as neonatal systems have improved.
- Preterm children are at a higher risk of developing ocular disorders, visual impairment, and amblyopia than term children.

What this study adds

- The magnitude and causes of ocular morbidity among preterm children are not well studied in sub-Saharan African countries. This study of preterm children admitted to two neonatal intensive care units in a sub-Saharan country shows that preterm infants develop a higher rate of visual impairment and amblyopia.

How this study might affect research, practice or policy

- The findings of this study provide some evidence for screening for ocular diseases in preterm children, but further studies are needed.
- A follow up prospective study commencing in 5 years' time would be of value as the number of surviving very low birth weight infants may significantly increase.

Confidential: For Review Only

INTRODUCTION

Global, regional, and national estimates of preterm birth (defined as childbirth at less than 37 completed weeks) using the 2019 Global Burden of Disease study showed 15.22 million preterm births (1). In the Global Burden of Disease Study, 3.1% of all disability-adjusted life-years were attributed to preterm birth, similar to the burden of HIV or malaria (2). More than 95% of preterm births are occurring in developing countries. Globally the estimated preterm birth rate is 11.1%. Over 60% of preterm births occur in Sub-Saharan Africa and South Asia (1). Ethiopia belongs in the top 15 countries that contribute to two-thirds of the world's preterm babies with a preterm rate of 14.1% out of 481 deliveries (3).

From six months of pregnancy to term is considered the most active period for ocular development (4). Improved neonatal care has increased the survival rates of extremely preterm infants with birth weights (BW) of 1,000g or gestational age (GA) of 28 weeks; at the same time, retinopathy of prematurity (ROP) has become a significant threat to visual function (5-7). Preterm children are reported to have an increased incidence of visual impairment because of perinatal lesions in the brain (8-10).

It has been noted that both preterm birth and ROP have an effect on the developing visual system, leading to decreased visual acuity, decreased contrast sensitivity, and an increase in color vision deficiencies (11-16). Population-based studies suggest that ophthalmic impairments remain common in very low birth weight infants (11,16,17). Effects of prematurity on ocular and neurological development include ROP, refractive error, strabismus, cerebral visual impairment, color vision deficits, reduced contrast sensitivity (CS), visual field defects, and decreased visual acuity (16). According to population studies, the incidence of ROP, whether moderate or severe, for infants born at less than 1500–1700g ranges from 22–49% (17-19).

In a cohort study, children with lower birth weights had significantly worse near and distance visual acuity at ages 10 to 12 years compared to full-term infants (10). Additionally, infants born prematurely without ROP are more likely to have myopia and anisometropia than infants born at term because preterm babies are more likely to experience refractive errors (20). An increased incidence of strabismus has also been reported in children born prematurely, regardless of the presence of ROP (21-24).

Confidential: For Review Only

Research on ocular morbidities among preterm infants in sub-Saharan African nations is limited. Before 2020, blindness from ROP was not reported in Ethiopia, including studies based on schools for the blind (25, 26).

To determine the top causes of illness and mortality in preterm infants admitted to neonatal intensive units (NICUs) in Ethiopia, an Ethiopian Study of Illness in Preterms (SIP) study was conducted based on standardized diagnostic protocols. This study is part of the SIP study focusing on ocular morbidities among preterms. The present study aimed to identify ocular disorders in a population of preterm children with and without ROP.

METHODS

Study design and subjects

The SIP Study is a prospective study conducted to determine the top causes of illness and mortality in preterm infants admitted to hospitals in Ethiopia based on standardized diagnostic protocols (27). The study participants of this current study are from the SIP study from Feb – June, 2019. The research was performed in accordance with the Declaration of Helsinki and was approved by the Institute Ethics Committee of Addis Ababa University ((Ref No. 003/2016). All parents or legal guardians provided informed consent before the examination. Patients or the public were not involved in research design, conduct, reporting, or dissemination plans.

Study setting

For the SIP study, standard protocols were developed to undertake a physical examination and laboratory investigation, in particular microbiology, radiologic, and ultrasound examinations. There were initial and follow-up examinations to detect the progress of the preterm infant. Addis Ababa University, Gondar University, Jimma University, and St. Paul Millennium Medical College were included in the SIP study. However, for this ocular morbidity aspect of the SIP study, only preterm children from Addis Ababa, Tikur Anbessa Hospital, Gandhi Hospital, and St. Paul Millennium Medical College were included.

Recruitment methods

Inclusion criteria were (1) GA < 37 weeks and (2) participation in the SIP study. The preterm children were identified from the SIP database. Parents of all preterm infants received a phone call invitation to participate in our investigation.

Confidential: For Review Only

Assessment of prenatal and postnatal history

History data were assessed from each child's recorded file for the enrolled children. The following antenatal risk factors were extracted: maternal age, in vitro fertilization, antenatal corticosteroids, preeclampsia/eclampsia, diabetes, HIV/AIDS, chorioamnionitis, mode of delivery, and multiple births. Neonatal factors extracted included sex, GA, BW, resuscitation in the delivery room, respiratory distress syndrome (RDS), duration of invasive/noninvasive mechanical ventilation and oxygen therapy, intracranial hemorrhage, patent ductus arteriosus (PDA), neonatal sepsis, necrotizing enterocolitis (NEC), number of blood transfusions, and bronchopulmonary dysplasia (BPD). There were no regular ROP screening programmes within the NICUs of the hospitals where the patients were admitted. There was no referral system from the NICUs to the Ophthalmology clinic, except if the parents noted a concern. In addition, all parents were interviewed using a standardized protocol to request information concerning medical history of the child and parents, including ocular and general morbidities.

Definitions

Gestational age was determined using last menstrual period [LPM], Ballard and Dubowitz scores and ultrasound assessment. Studies in Papua New Guinea have shown good concordance (0.878, 0.914, and 0.886, respectively) compared to antenatal ultrasound as the gold standard (28). LMP in a low-resource setting such as Bangladesh was found to be a more reliable measure of gestational age than previously thought for the estimation of postnatal gestational age of preterm infants (29).

Preterm infants were further classified as late and moderate preterm (32 to < 37 weeks), very preterm (28 to <32 weeks), and extremely preterm (less than 28 weeks). Glasses were prescribed if there was myopia >1.0D, astigmatism >1.0D, or hypermetropia >+2.0D.

Eye examination

All examinations were performed by the PI and lead author (STS), a pediatric ophthalmologist. Testing of best-corrected visual acuity was performed with Lea symbols until school enrolment, and after that, ETDRS was used in all subjects. In cases of visual acuity below 6/60, depending on the children's age, Lea symbols or Landolt rings were used at a distance of 1 m. Values were converted for analysis into the logarithm of the minimum angle of resolution (logMAR) (30).

https://mc.manuscriptcentral.com/bmjpo

Cyclopentolate (0.5%) eye drops were administered three times at 10-min intervals, after which cycloplegic refraction and keratometry were analyzed with an autorefractor (Nidek ARK-1s keratometer, Japan). The spherical equivalent refractive error was calculated by adding the spherical value and half of the cylindrical value. Anisometropia was defined as a difference between the patients' eyes of ≥ 1.5 diopters of spherical equivalent. Orthoptic examination for strabismus included the cover-uncover test and alternate cover test, the Hirschberg Test and examination of fixation behavior, as well as the presence or absence of nystagmus after having corrected refractive errors. If a child presented with heterotropia, an alternating prism cover test was added to measure the angle of deviation in prism diopters.

Strabismus was defined as constant or intermittent heterotropia of any dimension at a distance and/or near fixation after correcting refractive error. Classification of strabismus was categorized depending on deviation from the primary position (esotropia or exotropia). An anterior segment examination was done using slit lamp biomicroscopy. A dilated posterior segment examination was conducted using indirect ophthalmoscopy with a 28-diopter lens. Retinopathy of prematurity was diagnosed retrospectively from the patients' chart.

Data analyzed using IBM SPSS 21.0 (SPSS Inc., Chicago, USA). Continuous variables were expressed as the mean ± standard deviation (SD) or as the median when appropriate. Categorical variables were expressed as proportions. The chi-square test was used to analyze the association between categorical variables. Associations between ocular morbidities and continuous and categorical variables were computed using Fisher's exact test and Pearson chi-square (χ^2) test, respectively. Continuous variables were compared using ANOVA. Values of $p < 0.05$ were considered statistically significant.

For Review Only

RESULTS

During the study period 222 infants (146 from Saint Paul Hospital and 76 from TASH) were included in this study.

Characteristics of the study population

Slightly more females than males were screened (52.7% and 47.3%, respectively). The majority of the study participants (n=156, 70.3%) were less than 3 years of age and the mean age at presentation was 2.62 $\pm$ 0.49 years (range 2.08- 6.38). One hundred and twenty-three of the 222 children (55.4%) had a GA ≤34 weeks and 43 (19.4%) had a BW ≤1500g. Birthweight ranged from 953-3500g with a mean of 1941.72g (SD 445.49); GA ranged from 30-36 weeks, with a mean of 34.11 weeks (SD 1.47). One hundred and twenty-three children (55.4%) were delivered vaginally, and 80 (36.1%) had multiple gestations. Forty-eight children (21.7%) were born to mothers with pregnancy-induced hypertension, and eight (3.7%) mothers tested positive for HIV (Table 1).

The mean BWs of children from SPH and TASH NICUs were 1888.5 ± 403.6 (953-3000) g and 2043.94 ± 503.74 (1125-3500) g, respectively; mean GAs were 34.14 ± 1.49 (30-36) weeks and 34.08 ± 1.44 (30-36) weeks, respectively. Differences in these parameters were not statistically significant (Table 2).

Confidential: For Review Only

Table 1 Characteristics of premature children and mothers screened for ocular disorders

Variable		Total	Male		Female	
		N	N	%	N	%
Birthweight	≤ 1500g	43	20	46.5%	23	53.5%
	> 1500g	179	85	47.5%	94	52.5%
Gestational age	≤ 34 weeks	122	60	49.2%	62	50.8%
	> 34 weeks	100	45	45%	55	55%
Multiple gestation	Yes	80	42	52.5%	38	47.5%
	No	142	63	44.4%	79	55.6%
Oxygen supplementation	Yes	97	47	48.5%	50	51.5%
	No	125	58	46.4%	67	53.6%
Infantile morbidity	Sepsis	6	2	33.3%	4	66.7%
	IVH	2	0	0%	2	100%
	BPD and sepsis	1	1	100%	0	0%
	None	213	102	47.9%	111	52.1%
Mode of delivery	Vaginal delivery	123	53	43.1%	70	56.9%
	Cesarean section	99	52	52.5%	47	47.5%
Multiparity	Yes	47	18	38.3%	29	61.7%
	No	175	63	34.3	59	65.7%
Maternal morbidity	PIH	44	22	50%	22	50%
	HIV	5	2	40%	3	60%
	HIV & PIH	3	3	100%	0	0%
	DM	2	0	0%	2	100%
	DM & PIH	1	0	0%	1	100%
	TORCH	1	1	100%	0	0%
	None	166	77	46.4%	89	53.6%
NICU location	SPH	146	65	44.5%	81	55.5%
	TASH	76	40	52,6%	36	47.4%

Legend: BPD- Bronchopulmonary Dysplasia; DM- Diabetes Mellitus; HIV-IVH-Intraventricular Hemorrhage; PIH-Pregnancy-Induced Hypertension; SPH- Saint Paul Hospital; TASH-Tikur Anbessa Specialized Hospital; TORCH-Toxoplasmosis, Rubella, Cytomegalovirus, Herpes simplex, and HIV

Confidential: For Review Only

Ocular morbidities and risk factors

Overall, 145 (65.3%) of the children had ocular disorders at the presentation, of which 92 (63.4%) had isolated ocular diseases (69 refractive error, 13 nasolacrimal duct obstruction, five strabismus, and five ROP). The mean age at presentation of children with ocular disorders was 2.7±0.5 (2.1- 6.4) years, and there were more females with a male to female ratio of 1:1.27. None of the eyes examined had anomalies of the anterior segment or lens.

The mean GA was 34.14 ± 1.49 (30-36) weeks, and BW was 1927.27 ± 429.19 (953-3100) g. Refractive errors were the leading type of ocular morbidity seen in 115/222 (51.8%), followed by NLDO (21.2%) (Table 2).

Table 2. Types of ocular disorders among premature children screened

Ocular disorders	n	%
Refractive error	115	51.8
Nasolacrimal duct obstruction	47	21.2
Strabismus	25	11.3
Retinopathy of prematurity	16	7.2
Others	5	2.3

NB- Some ocular disorders occurred more than once.

Refractive error

One hundred and fifteen (51.8%) children had a refractive error, of which 55.5% (81/146) and 44.7% (34/76) of children enrolled from the SPH and TASH had refractive errors, respectively. The mean age at presentation was 2.68 ±0.56 (2.08-6.38) years, and the male-to-female ratio was 1:1.25. Thirty-nine (59%) of children aged > 3 years developed refractive error in comparison with 76 (48.1%) of those aged < 3 years.

The mean gestational age and birthweight of children with refractive errors was 34.11±1.54 (30-36) weeks and 1892.34±414.55 (1080-3100) g, respectively. Myopia was the commonest type of refractive error, accounting for 78/115 (60.8%) of cases, followed by astigmatism (30, 26.1%) and hyperopia (15, 13.1%). Gender, GA, BW, oxygen supplementation, children, and maternal morbidity were not statistically associated with refractive error. (Table 3)

Strabismus

Twenty-five children (11.3%) had strabismus (5 isolated, 20 in combination with refractive error, nystagmus, ROP, and nasolacrimal duct obstruction). The age at presentation was 2.73±0.52 (2.1-3.6) years, and the male-to-female ratio was 1.08:1.

The mean GA and BW were 34.0±1.41 (30-36) weeks and 1906.76±489.92 (1140-3000) g, respectively. Thirteen children had esotropia, and the rest had exotropia. There was no statistically significant association between GA, BW, and strabismus (Table 3). In this study, the prevalence of strabismus among children aged ≥ 3years was 16.7% compared to 8.9% in those < 3 years. However, older age was not statistically associated with strabismus.

Confidential: For Review Only

Retinopathy of prematurity

Previous history of ROP was noted in 16/222 (7.2%) of the children enrolled in this study. Most patients (12, 57%) with ROP were from SPH. Almost all of them (15/16) had a GA < 34 weeks, and the mean GA and BW of patients with ROP were 32.19±1.33 (30-35) weeks and 1596.25 ±483.64 (953-2600) g, respectively. Nine patients with ROP had an associated refractive error (6 myopia and 3 astigmatism). Only one patient had an associated intermittent exotropia.

In univariate analysis, ROP was statistically associated with low GA and low BW (Table 3). Multivariable logistic regression analysis was not conducted due to the small number of children with ROP.

Table 3. Ocular disorders by sex, gestational age, and birthweight among premature children screened for ocular disorders

Type of Disorder	Variables		Yes	No	Odds Ratio	p-Value
			n	n		
Refractive error	Sex	Male	51	54	0.78(0.46-1.33)	0.361
		Female	64	53		
	BWt	≤1500 gm	22	21	1.03(0.53-2.01)	0.926
		>1500gm	93	86		
	GA	≤34 weeks	62	60	1.09(0.64-1.85)	0.746
		> 34 weeks	53	47		
Strabismus	Sex	Male	12	93	1.03(0.4501.37)	0.940
		Female	13	104		
	BWt	≤1500 gm	7	36	0.57(0.22-1.48)	0.246
		>1500gm	18	161		
	GA	≤34 weeks	14	108	0.95 (0.41-2.2)	0.911
		> 34 weeks	11	89		
ROP	Sex	Male	6	99	0.65(0.22-1.85)	0.415
		Female	10	107		
	BWt	≤1500 gm	7	36		0.01
		>1500gm	9	170	0.27(0.09-0.78)	
	GA	≤34 weeks	15	107		0.001
		> 34 weeks	1	99	0.72 (0.09-0.56)	

Legend: BWt- Birth Weight ; GA-Gestational Age

Visual impairment and ocular disorders

The mean VA of the right and left eyes was 0.22(SD 0.23) logMAR and 0.17 (SD 0.21) logMAR, respectively. The mean VA in the better and worse eyes was 0.17 (SD 0.22) logMAR and 0.28 (SD 0.21) logMAR, respectively. In this study, 101 (45.9%) and 181 (81.5%) of the children had subnormal visual acuity (>logMAR 0.1) in the better and worst eyes, respectively.

Confidential: For Review Only

Nearly one-fourth (55, 24.8%) of children screened had visual impairment in the better eye. Of this group 51 (92.7%) had uncorrected refractive error alone (34/51) or with strabismus (10/51), ROP (6/51), or nystagmus (1/51). Eighty-nine (40.1%) patients had amblyopia, of which 59/89 (66.3%) had bilateral amblyopia from uncorrected refractive error. Isometropic and anisometropic amblyopia from uncorrected refractive error were the commonest causes of amblyopia, contributing to 49/89 (55%) and 20/89 (22.8%) of cases, respectively. Of the 16 cases with ROP, 12 (75%) had a visual impairment associated with other disorders like refractive error, strabismus, and nystagmus.

In univariate analysis, visual impairment in the better eye was statistically associated with ROP, uncorrected refractive error, and strabismus with p-values of 0.001, 0.001, and 0.004, respectively. Amblyopia was not statistically associated with low GA or low BW (Table 4).

Table 4. The presence of visual impairment by types of ocular disorders among premature children screened for ocular disorders.

**Variables**		**Visual impairment in the better eye**		**Odds ratio 95% CI (Lower-Upper)**	**p-Value**
		Yes	No		
**Sex**	Male	23	82	0.75(0.40-1.38)	0.348
	Female	32	85		
**BWt**	≤ 1500g	14	29	0.62(0.29-1.27)	0.188
	> 1500g	41	138		
**GA**	≤ 34 weeks	28	94	1.24(0.67-2.29)	0.294
	> 34 weeks	27	73		
**Refractive error**	Yes	48	67	10.24(4.37-23.97)	0.001
	No	7	100		
**Strabismus**	Yes	12	13	3.31 (1.41-7.77)	0.004
	No	43	154		

Confidential: For Review Only

DISCUSSION

The present prospective study examines the effects of prematurity on visual acuity and ocular disorder in children born preterm. In Sub-Saharan Africa, neonatal death has decreased by 40% since 1990 due to improved newborn care, likely leading to an increase in childhood ocular morbidity and blindness from diseases like ROP (32). Despite this positive progress, data on the extent of ocular diseases among the preterms in Sub-Saharan Africa are limited. Our study has demonstrated that the prevalence of ocular diseases and visual impairment in Ethiopian children born preterm is high. To our knowledge, this is the first study to assess the prevalence and causes of ocular disorders and visual impairment among children born preterm and admitted to NICUs.

In Ethiopia, intensive neonatal care has expanded in many public and private hospital NICUs since 2013 (33), and neonatal mortality per thousand live births has declined modestly from 39 in 2000 to 33 in 2019(34). A prospective screening survey among neonates admitted to two NICUs in Ethiopia showed that 32.2% of the screened infants had any stage ROP (35). However, there is no regular ROP screening program in the country. A comparison of studies of ocular morbidity and visual impairment among preterm children is difficult as there are methodological variations such as differing age groups, inclusion or exclusion of ROP, stages of ROP, and cohort size.

Even though genetic and visual experiences predominantly determine the prevalence of refractive error, studies have shown that low BW interrupts emmetropisation and increases the prevalence of refractive error (36). In our study, nearly half of the premature screened children (51.8%) had refractive error, which is comparable to a survey from Turkey (53.8%) (37) but higher than in Italy (42.3%) (38), and in cohorts of extremely preterm infants from Sweden (29.7%) (39) and Norway (10%) (40). In our study, the prevalence of myopia was 35.1%, which was higher than a cohort of preterm children at age 10–12 years from the UK (18.9%) (41), India (15.8%) (42), and Sweden (4.1%) (39). The prevalence of hyperopia in our study, 13.5%, is comparable with that reported in Turkey (14.3%) (37) and Sweden (17.1%) (39) but higher than the UK (6.6%) (41) and India (8.54%) (42). In the present study, 13.5% of the preterm children had clinically significant astigmatism, which was lower than that reported in Norway (21%) (40) and India (55.6%) (42) and higher than in Turkey (5.7%) (37) and Sweden (6.5%) (39). The higher proportion of myopia seen in our study, in comparison with studies from the UK (41),

Confidential: For Review Only

Confidential: For Review Only

India (42) and Sweden (39), is supported by long-term studies which have confirmed the increased incidence of myopia following preterm birth (43).

Manifest strabismus was seen in 11.3% of our cohort, which is comparable to studies from Norway (10%) (40), the UK (13.6%) (15), Sweden (13.5%) (20), and Australia (14%) (45), and lower than reported in Sweden (17%) (36), the UK and Ireland (24%) *(46)* and Germany (26%) (47). It is unclear at what age the different types of strabismus develop (36), and the age at onset of strabismus in low birthweight children is variable, from the first few months of life to many years later (11, 15, 16,21,22, 44). In our study, a higher prevalence of strabismus in those aged > 3 years was noted. This finding (16.7%) is comparable with a similar age group from Sweden (20). Regarding the type of strabismus, we detected similar proportions for esotropia and exotropia. This is similar to the other studies from Germany (47) and England (41). However, other investigations confirmed that esotropia was the most frequent type of strabismus (20, 39, 48) The increased prevalence of strabismus in the low birthweight population is well-documented (21, 36, and 44). Such an association was not apparent in our study, as most of the children were considerably higher in weight and older than in the studies mentioned above.

The prevalence of ROP in our study is 7.2%, lower than in other studies, from sub-Saharan African countries, including Ethiopia, which ranged from 15-41.7% (35, 49-51). The lower prevalence of ROP in our study can be explained by our data collection method, where we depend on the history of ROP either from the patient's parents or from old features of ROP.

In the present study, 46% of the children had subnormal visual acuity (>logMAR 0.1) in the better eye, which is comparable with a population-based study from Norway (45.9%) (40). The figure is higher than what has been reported for prematurely-born children with BWs 1500–2000 g (15 %) from Denmark (9) and from Sweden 32 % (20). Birch et al. reported significantly lower visual acuities in low birthweight infants compared to those born full term (52). In our study, there was no statistically significant correlation between BCVA and BW or GA, similar to a study from Turkey (37). However, Dowdeswell et al. (53) found low levels of distance visual acuity in preterm children compared with full-term children. However, in our study, ocular morbidities like strabismus, refractive error, and ROP were statistically associated with visual impairment.

In our study, the prevalence of amblyopia among premature children was 40.1%. The result in our study is much higher than other studies from Australia (7.3%) (45) and Turkey

(7.7%) (37). Previous studies have shown that prematurity and low birthweight are two risk factors for amblyopia (41, 54). Nevertheless, amblyopia was not statistically associated with low GA and BW. Even if we did not find a statistical association between GA and BW with amblyopia, the prevalence among premature children is higher than in other studies; this indicates that more importance should be given to screening amblyopia risk factors for premature infants.

The strengths of this study were the prospective controlled study design with a high number of participants, the multi-center design which increases the representativeness of our research, and the availability of medical information from all children and mothers, which allowed a very detailed examination and an adjustment for different possible confounding factors. The strict standardization reduced the probability of examiner-dependent variances.

Limitations of the study included the wide age range of the examined children, some of whom were at an early age and phase of refractive development, and other older children that can affect the physiologic refractive changes noted in normal health children. The other limitation is there is a chance that those infants with poor health outcomes did not take part in our study. In subsequent research, we will continue following up with these infants to determine future changes in their refractive error and strabismus.

CONCLUSION

In conclusion, the rates of ocular disorders, visual impairment, and amblyopia in these NICUs in Ethiopia were higher than in other studies. Refractive error, strabismus, and ROP were all significant risk factors for visual impairment. These findings underline the importance of early screening of premature infants for vision and amblyopia. As the two NICUs included in the survey are Ethiopia's main neonatal referral centers, it can be postulated that ocular morbidities, visual impairment, and amblyopia are emerging as potentially avoidable causes of childhood blindness among preterm children in Ethiopia. Developing preterm ocular-related screening protocols within the NICUs, strengthening the referral links between the NICUs and eye centers, and further detailed comparative studies between preterm and term children for ocular disorders are recommended.

Confidential: For Review Only

Lists of Abbreviations

Bronchopulmonary dysplasia (BPD); Necrotizing enterocolitis (NEC); Neonatal Intensive Care Units (NICUs); Patent ductus arteriosus (PDA); Preterm birth (PTBs); Respiratory distress syndrome (RDS); Retinopathy of prematurity (ROP), Saint Paul Hospital (SPH) and Tikur Anbessa Specialized Hospitals (TASH).

Research Ethics Approval

This study involves human participants, and this research was approved by the Addis Ababa University Ethics Review Committee (Ref No. 003/2016) in line with the relevant national and institutional guidelines on care and clinical research. All parents or legal guardians gave written informed consent before participating in the study.

Availability of Data and Materials

All data generated or analysed during this study are included in this published article

Competing Interests

The authors have no conflicts of interest.

Funding

The SIP study was supported by the Bill & Melinda Gates Foundation (OPP1136965)

Authors' Contributions

Drafting of the manuscript: STS., LM., AM. , and AD. Revision of the manuscript for important intellectual content: STS., LM., AM., AD and AA. Conception and design of study: STS., LM., AM., and AD. Data acquisition, analysis, or interpretation of data: STS., LM., AM., AD., and AA. Approval of final manuscript to be published: STS., LM., AM., AD., and AA. All authors have read and approved the final version of the manuscript.

Acknowledgements

The authors wish to acknowledge the assistance of the staff of the SIP project (Ahmed, Beleyu, Efrata, and Wagaye) for their support during data collection. Our special appreciation goes to Sr Martha H/Mariam and Sr. Medhanit Tesfaye from Menelik II pediatrics Ophthalmology clinic.

Confidential: For Review Only

REFERENCES

1. Cao G, Liu J, Liu M. Global, regional, and national incidence and mortality of neonatal preterm birth, 1990-2019. JAMA pediatrics. 2022 Aug 1;176(8):787-96.

2. World Health Organization. 2014. Global Burden of Diseases Estimates http://www.who.int/healthinfo/global_burden_disease/en/.

3. Adane AA, Ayele TA, Ararsa LG, Bitew BD, Zeleke BM. Adverse birth outcomes among deliveries at Gondar University Hospital, Northwest Ethiopia, BMC Pregnancy and Childbirth 2014, 14:90.

4. Hellström A, Smith LE, Dammann O. Retinopathy of prematurity. The lancet. 2013 Oct 26;382(9902):1445-57.

5. Gogate P, Gilbert C, Zin A. Severe visual impairment and blindness in infants: causes and opportunities for control. Middle East Afr J Ophthalmol. 2011;18(2):109–114.

6. Chen C, Zhang QS. [Advances in medical care for extremely low birth weight infants worldwide]. Zhongguo Dang Dai Er Ke Za Zhi. 2013;15(8):703–707. Chinese.

7. Mccolm JR, Fleck BW. Retinopathy of prematurity: causation. Semin Neonatol. 2001;6(6):453–460.

8. Cats BP, Tan KEWP. Prematures with and without regressed retinopathy of prematurity: comparison of long-term (6–10 years) ophthalmological morbidity. J Pediatr Ophthalmol Strab 1989;Nov/Dec:271–5.

9. Fledelius HC, Greisen G. Very pre-term birth and visual impairment. Acta Ophthalmol 1993;Suppl 210:63–5.

10. Gallo JE, Lennerstrand G. A population-based study of ocular abnormalities in premature children aged 5 to 10 years. Am J Ophthalmol 1991;111:539–47.

11. Holmstrom G, el Azzazi M, Kugelberg U. Ophthalmological follow up of preterm infants: a population based, prospective study of visual acuity and strabismus. Br J Ophthalmol 1999;83:143–50.

12. Sebris SL, Dobson V, Hartmann EE. Assessment and prediction of visual acuity in 3-to 4-year-old children born prior to term. Human Neurobiol 1984;3:87–92.

13. Visual function in low birthweight children A R O'Connor, T J Stephenson, A Johnson, M J Tobin, S Ratib, M Moseley, A R Fielder.

Confidential: For Review Only

14. Larsson E, Rydberg A, Holmstrom G. Contrast sensitivity in 10 year old preterm and full term children: a population basedstudy. Br J Ophthalmol 2006;90:87–90.
15. Cooke RW, Foulder-Hughes L, Newsham D, Clarke D. Ophthalmic impairment at 7 years of age in children born very preterm. Arch Dis Child Fetal Neonatal Ed 2004;89:F249–53.
16. O'Connor AR, Stephenson TJ, Johnson A, Tobin MJ, Ratib S, Moseley M, et al. Visual function in low birthweight children. Br J Ophthalmol 2004;88:1149–53.
17. Darlow BA, Clemett RS, Horwood LJ, et al. Prospective study of New Zealand infants with birth weight less than 1500 g and screened for retinopathy of prematurity: visual outcome at age 7–8 years. Br J Ophthalmol 1997;81:935–40.
18. Ng YK, Fielder AR, Shaw DE, Levene MI. Epidemiology of retinopathy of prematurity. Lancet 1988; 2(8622): 1235–1238.
19. Holmstrom G, el Azazi M, Jacobson L, Sachs D, Sule J, Lennerstrand G. Epidemiology of ROP in the Stockholm area of Sweden. Acta Opthalmol 1993; 210(Suppl): 44–47.
20. Holmström G, El Azazi M, Kugelberg U. Ophthalmological long term follow up of preterm infants: a population based, prospective study of the refraction and its development. Br J Ophthalmol. 1998;82:1265–1271.
21. Laws D, Shaw DE, Robinson J, et al. Retinopathy of prematurity: a prospective study. Review at six months. Eye 1992;6:477–83.
22. Pennefather PM, Clarke MP, Strong NP, et al. Ocular outcome in children born before 32 weeks gestation. Eye 1995;9(Suppl):26–30.
23. Robinson R, O'Keefe M. Follow-up study on premature infants with and without retinopathy of prematurity. Br J Ophthalmol 1993;77:91–4.
24. McGinnity FG, Bryars JH. Controlled study of ocular morbidity in school children born preterm. Br J Ophthalmol 1992;76:520–4.
25. Asferaw M, Woodruff G, Gilbert C. Causes of severe visual impairment and blindness in students in schools for the blind in Northwest Ethiopia. BMJ Glob Health. 2017;2(2):e000264.
26. Kello AB, Gilbert C. Causes of severe visual impairment and blindness in children in schools for the blind in Ethiopia. Br J Ophthalmol. 2003;87(5):526-30.
27. Muhe, L.M., McClure, E.M., Mekasha, A. et al. A Prospective Study of Causes of Illness and Death in Preterm Infants in Ethiopia: The SIP Study Protocol. Reprod Health 15, 116 (2018). https://doi.org/10.1186/s12978-018-0555-y.

Confidential: For Review Only

28. Karl S. Connie SN, Wai Suen et al. 2015. Preterm or Not – An Evaluation of Estimates of Gestational Age in a Cohort ofWomen from Rural Papua New Guinea. PLOSONE |DOI:10.1371/journal.pone.0124286 May6, 2015.

29. Rosenberg RE, Nawshad ASM, Ahmed U. et al 2009. Determining Gestational Age in a Low-resource Setting: Validity of Last Menstrual Period. J HEALTH POPUL NUTR 2009 Jun;27(3):332-338.

30. Bach M, Kommerell G. Determining visual acuity using European normal values: scientific principles and possibilities for automatic measurement. Klin Monatsbl Augenheilkd. 1998;212(4):190–5.

31. Hosmer DW, Lemesbow S. Goodness of fit tests for the multiple logistic regression model. Communications in statistics-Theory and Methods. 1980 Jan 1;9(10):1043-69.

32. Hug L, Alexander M, You D, Alkema L. National, regional, and global levels and trends in neonatal mortality between 1990 and 2017, with scenario-based projections to 2030: a systematic analysis. The Lancet Global Health. 2019 Jun 1;7(6):e710-20.

33. UNICEF. Investing in Survival: Enhancing the Neonatal Intensive Care Unit of Yekatit 12 Hospital: A Final Report for UNICEF's Next Generation; 2013 file. https://www.unicefusa.org/sites/default/files/Ethiopia%20Report.pdf. Accessed January 2023.

34. Ministry of Health-Ethiopia. Health Sector Transformation Plan-II. 2021. https://www.globalfinancingfacility.org/ethiopia-health-sector-transformation-plan-201920-202425. Accessed January 2023.

35. Sherief ST, Taye K, Teshome T, Demtse A, Gilbert C. Retinopathy of prematurity among infants admitted to two neonatal intensive care units in Ethiopia. BMJ Open Ophthalmology. 2023 Jul 1;8(1):e001257.

36. O'Connor A, Fielder AR. Long term ophthalmic sequelae of prematurity. Early human development. 2008 Feb 1;84(2):101-6.

37. Özdemir M, Koylu S. Ocular growth and morbidity in preterm children without retinopathy of prematurity. Japanese journal of ophthalmology. 2009 Nov;53:623-8.

38. Ricci B. Refractive errors and ocular motility disorders in preterm babies with and without retinopathy of prematurity. Ophthalmologica. 1999 Oct 1;213(5):295-9.

39. Hellgren KM, Tornqvist K, Jakobsson PG, Lundgren P, Carlsson B, Källén K, Serenius F, Hellström A, Holmström G. Ophthalmologic outcome of extremely preterm infants at 6.5 years

Confidential: For Review Only

of age: Extremely Preterm Infants in Sweden Study (EXPRESS). JAMA ophthalmology. 2016 May 1;134(5):555-62.

40. Haugen OH, Nepstad L, Standal OA, Elgen I, Markestad T. Visual function in 6 to 7 year-old children born extremely preterm: a population-based study. Acta ophthalmologica. 2012 Aug;90(5):422-7.

41. O'Connor AR, Stephenson TJ, Johnson A, Tobin MJ, Ratib S, Fielder AR. Change of refractive state and eye size in children of birth weight less than 1701 g. British journal of ophthalmology. 2006 Apr 1;90(4):456-60.

42. Sathar A, Abbas S, Nujum ZT, Benson JL, Sreedevi GP, Saraswathyamma SK. Visual outcome of preterm infants screened in a tertiary care hospital. Middle East African Journal of Ophthalmology. 2019 Jul;26(3):158.

43. O'Connor AR, Wilson CM, Fielder AR. Ophthalmological problems associated with preterm birth. Eye. 2007 Oct;21(10):1254-60.

44. Theng JT, Wong TY, Ling Y. Refractive errors and strabismus in premature Asian infants with and without retinopathy of prematurity. Singap Med J 2000;41:393–7.

45. Hebbandi SB, Bowen JR, Hipwell GC, Ma PJ, Leslie GI, Arnold JD. Ocular sequelae in extremely premature infants at 5 years of age. Journal of paediatrics and child health. 1997 Aug;33(4):339-42.

46. Marlow N,Wolke D, BracewellMA, SamaraM; EPICure Study Group. Neurologic and developmental disability at six years of age after extremely preterm birth. N Engl J Med. 2005;352(1):9-19.

47. Fieß A, Kölb-Keerl R, Schuster AK, Knuf M, Kirchhof B, Muether PS, Bauer J. Prevalence and associated factors of strabismus in former preterm and full-term infants between 4 and 10 Years of age. BMC ophthalmology. 2017 Dec;17(1):1-9.

48.VanderVeen DK, Bremer DL, Fellows RR, Hardy RJ, Neely DE, Palmer EA, Rogers DL, Tung B, Good WV, Early Treatment for Retinopathy of Prematurity Cooperative G. Prevalence and course of strabismus through age 6 years in participants of the early treatment for retinopathy of prematurity randomized trial. J AAPOS. 2011;15(6):536–40.

49. Fajolu IB, Rotimi-Samuel A, Aribaba OT, et al. Retinopathy of prematurity and associated factors in Lagos, Nigeria. Paediatr Int Child Health. 2015;35(4):324-8.

Confidential: For Review Only

50. Omer IM, Hassan HA. The prevalence and risk factors of retinopathy of prematurity among preterm babies admitted to Soba Neonatal Intensive Care Unit. Sudan J Paed. 2014;14(2):17.

51. Wanjala I ID, Kariuki L. Retinopathy of Prematurity as seen in two major hospitals in Nairobi, Kenya. East Afr J Ophthalmol. 2007 May;13(1):5-14.

52. Birch EE, O'Connor AR. Preterm birth and visual development. InSeminars in Neonatology 2001 Dec 1 (Vol. 6, No. 6, pp. 487-497). WB Saunders.

53. Dowdeswell HJ, Slater AM, Broomhall J, Tripp J. Visual defi cits in children born at less than 32 weeks gestation with and without major ocular pathology and cerebral damage. Br J Ophthalmol 1995;79:447–452.

54. Mocanu V, Horhat R. Prevalence and risk factors of amblyopia among refractive errors in an Eastern European population. Medicina. 2018 Mar 20;54(1):6.

Confidential: For Review Only

TABLE LEGEND

Table 1 Characteristics of premature children and mothers screened for ocular disorders

Table 2. Types of ocular disorders among premature children screened

Table 3. Ocular disorders by sex, gestational age, and birthweight among premature children screened for ocular disorders

Table 4. The presence of visual impairment by types of ocular disorders among premature children screened for ocular disorders.

Confidential: For Review Only
